# A computational model of inner speech supporting flexible goal-directed behaviour in Autism

**DOI:** 10.1038/s41598-022-18445-9

**Published:** 2022-08-20

**Authors:** Giovanni Granato, Anna M. Borghi, Andrea Mattera, Gianluca Baldassarre

**Affiliations:** 1 Laboratory of Computational Embodied Neuroscience, Institute of Cognitive Sciences and Technologies, National Research Council of Italy, Rome, Italy; 2 School of Computing, Electronics and Mathematics, University of Plymouth, Plymouth, UK; 3 Dipartimento di Psicologia Dinamica, Clinica e Salute, Sapienza University of Rome, Institute of Cognitive Sciences and Technologies, National Research Council of Italy, Rome, Italy

**Keywords:** Cognitive ageing, Computational neuroscience, Sensory processing, Autism spectrum disorders, Human behaviour

## Abstract

Experimental and computational studies propose that inner speech boosts categorisation skills and executive functions, making human behaviour more focused and flexible. In addition, many clinical studies highlight a relationship between poor inner-speech and an executive impairment in autism spectrum condition (ASC), but contrasting findings are reported. Here we directly investigate the latter issue through a previously implemented and validated computational model of the Wisconsin Cards Sorting Tests. In particular, the model was applied to explore potential individual differences in cognitive flexibility and inner speech contribution in autistic and neurotypical participants. Our model predicts that the use of inner-speech could increase along the life-span of neurotypical participants but would be reduced in autistic ones. Although we found more attentional failures (i.e., wrong behavioural rule switches) in autistic children/teenagers and more perseverative behaviours in autistic young/older adults, only autistic children and older adults exhibited a lower performance (i.e., fewer consecutive correct rule switches) than matched control groups. Overall, our results corroborate the idea that the reduced use of inner speech could represent a disadvantage for autistic children and autistic older adults. Moreover, the results suggest that cognitive-behavioural therapies should focus on developing inner speech skills in autistic children as this could provide cognitive support throughout their whole life span.

## Introduction

Humans commonly use inner speech, a covert and self-directed form of language without motor articulation^1^. Evidence has shown that inner speech plays an important role in supporting cognition. In particular, it boosts categorisation processes^2–4^, executive functions^5–7^, working memory^8^, metacognition^9^, and motivation^10^.

Several experimental and clinical studies started to investigate the role of inner speech in neurodevelopmental and psychiatric conditions^11^. These studies show that inner speech can generally provide cognitive support, but in some cases it can also have disruptive effects. For example, in schizophrenic patients it can be distracting, fragmented, charged with negative emotions, and possibly involve auditory hallucinations.

Here we focus on the Autism Spectrum Condition (ASC), a condition characterised by repetitive behaviours, social impairment, sensory alterations, and restricted interests (we refer to DSM-V for diagnostic criteria^12^). Specifically, we focus on a sub-set of spectrum conditions that do not show comorbidity with an intellectual disability. There is an open debate regarding the use of the terms ‘condition’ or ‘disorder’ to refer to Autism^13^. Here we use the term ‘condition’ to avoid stigma without ignoring the daily challenges and possible impairments it involves.

Many studies investigated the relationship between inner speech and executive functions in Autism, and contrasting results are reported (for a review see^14^). For example, some studies on planning^15–17^ found that an experimental interference of inner speech (e.g., articulatory suppression) impairs planning abilities in control participants but not in an autistic sample . However, the results should be taken with caution due to potential methodological limitations (see critiques by^14^). Again, evidence on working memory suggests that autistic individuals do not spontaneously use inner speech to name stimuli internally (e.g.^18^) while most studies on motor control indicate either that autistic individuals use inner speech or that the absence of inner speech does not impact their performance. Crucially for us, Russell-Smith et al.^19^ showed that articulatory suppression does not interfere with cognitive flexibility in autistic people, who do not show an impaired performance. However, Winsler et al.^20^ found that autistic children performed worse than controls even if they did used use private speech. Overall, these scattered and controversial results leave space to further research. In particular, findings suggest that autistic people make reduced use of inner speech but it is debated whether this reduction has an impact on executive functioning and in particular on cognitive flexibility.

The aim of this work is to investigate the relationship between inner speech and executive functions in autistic people. In particular, we aim to propose interpretations and predictions that inform about the potential differences between autistic and non-autistic people. Specifically, we have used here a previously implemented and validated computational model^7^ able to perform the Wisconsin Cards Sorting test (WCST), a neuropsychological test commonly used to measure cognitive flexibility^21^. The model is capable of reproducing several human behavioural data, also distinguishing the different levels of inner speech contribution during the administration of a verbal shadowing protocol. Here we use the model to address four studies in which the WCST is administered to autistic children^22^, teenagers^23^, young adults^24^ and middle adults^25^. According with literature interpretations^26,27^, our model predicts that the control groups would show an inner speech contribution that progressively increases with the age of the participants. Instead, autistic people would show a reduced inner speech contribution at any age. Moreover, autistic children and middle adults show statistically significant lower global performances than their control groups. While acknowledging that this work considers an experimental setting and hence has limited ecological validity^28^ , these results can have clinical implications. In particular, they suggest focusing psychotherapies treatments on the development of inner speech skills, especially in autistic children. Adequate inner language support could be a protective factor against ageing in autistic people, compensating for age-dependent physiological cognitive decline.

## Methods

### Task and participants data

**Figure 1:**
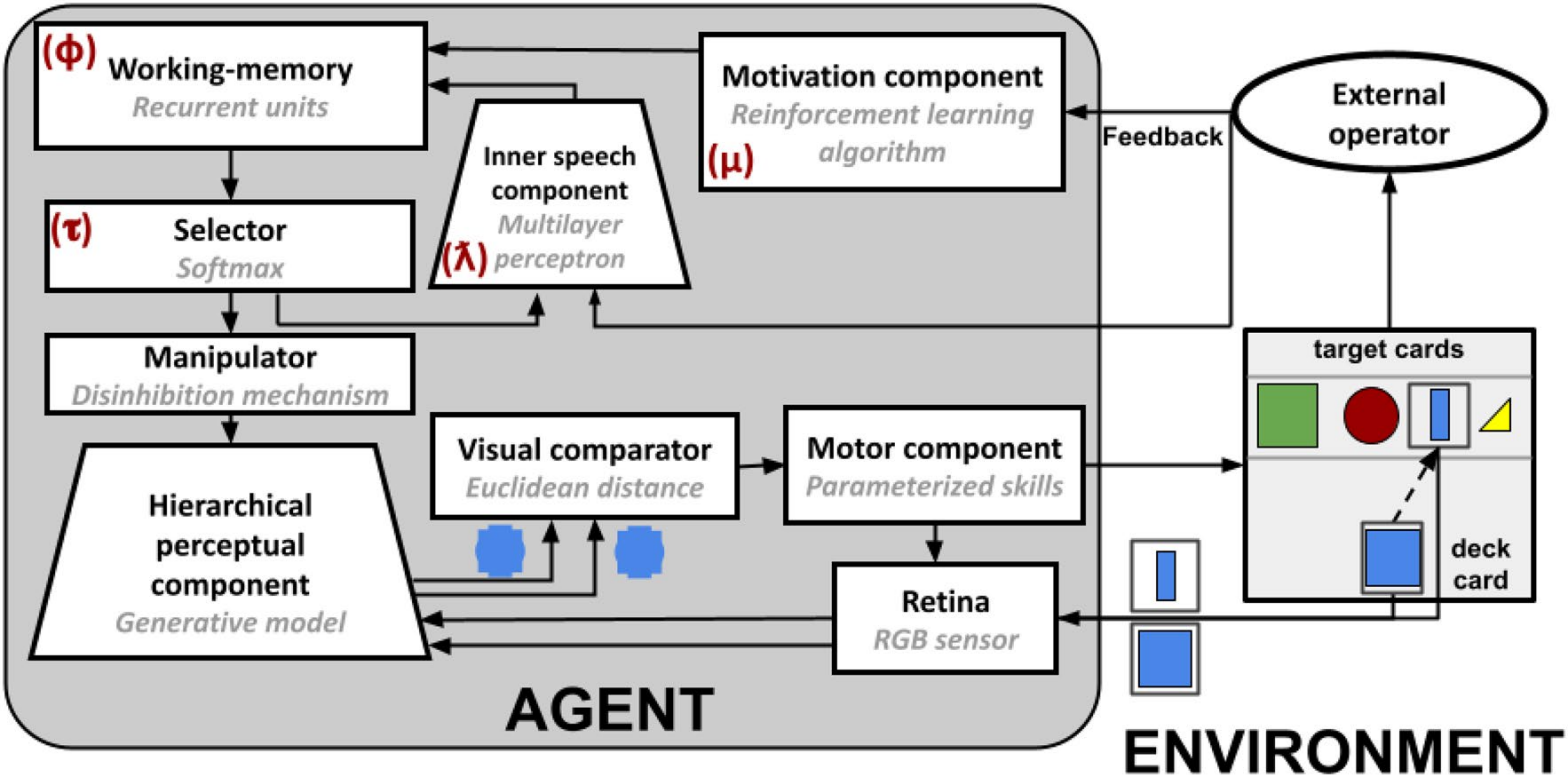
Architecture of the model. In each box, the component label is highlighted in bold, and the computational algorithm implemented by the component is highlighted in italic grey. The Greek symbols in red indicate the four key parameters of the model. The two little blue images under the visual comparator highlight that the visual comparison is based on the low-level representations of the deck and selected target cards. These visual representations are produced by the hierarchical component, through its generative capabilities, on the basis of the selected-rule top-down bias (here ‘colour’).

The WCST^29^ is a neuropsychological test that is commonly used to measure cognitive flexibility, the capacity to change behavioural strategies to achieve a target goal on the basis of external feedback^21^. The task setting is composed of two decks of 64 cards and four target cards, put on a table in front of the participant (Fig. 1). Each deck card shows a specific combination of elements varying with respect to three categories (colour, shape, number), each characterised by four attributes (colour: red, green, blue, yellow; shape: stars, triangles, circles, crosses; number: one, two, three, four). Each target card shows a unique combination of attributes (e.g., one red triangle, two green circles, etc.). In our simulations the third category (i.e., numerosity) is substituted with size (small, medium small, medium large, and large), and the star and cross shapes are substituted with square and bar shapes. We made these changes to reduce computational resource requirements and to gain simulation speed. In particular, the presence of many items (i.e., numerosity rule) and more complex images (e.g., stars) would require a higher sensory resolution and therefore more computational resources. As we demonstrated in^6,7^ these modifications do not alter the quality of the results, still allowing the reproduction of human behavioural data. Note that this result agrees with theories and models on rule-based category learning^6,30^, suggesting that it is mostly based on a rule/representations switch process. In particular, rule-based category learning should not depend on specific categories/rules or shapes, provided that the participant has correctly acquired abstract and perceptual representations of them (we begin to investigate the representations learning issue in^31^). Participants are required to sort each deck card, choosing one of the three categories. After the category selection they have to put the drawn deck card under one target-card sharing the attribute of the chosen category. For example, if the chosen category is ‘colour’, a card with a blue item has to be put under the target card with a blue item. Importantly, there is a correct sorting rule for each turn, but it is unknown to the participant. After each sorting attempt, an external operator provides feedback (‘correct’ or ‘not correct’) depending on the current sorting rule and the action executed. The key challenge of the task is thus that the participant has to infer the rule based on the feedback. After a succession of ten correct matches, the sorting rule changes without informing the participant, who has thus to change the sorting rule to the new one by inferring it on the basis of the feedback.

To extract a complete cognitive profile of participants, we have considered the following four behavioural indices scored during the task performance. *Completed Categories* (CCs), identifying the number of ten-cards sorting sequences performed without an error. This index reflects the global performance. *Perseverative Errors* (PEs), identifying the number of times in which a participant chooses an incorrect previous chosen rule. This behavioural error indicates a perseverative behaviour. *Non Perseverative Errors* (NPEs), identifying all errors that do not fall into the previous ones. These behavioural errors indicate an attentional failure or incorrect inferential reasoning. At last, *Failure-to-maintain Set* (FMS), identifying a premature and incorrect rule change (i.e. performed before a successful ten-cards sorting sequence is reached). This behavioural error indicates a sustained attention failure.

To investigate the relationship between flexible behaviour and inner-speech in Autism we performed a selection of four experimental studies. This process is the product of an extensive review process that has highlighted many shortcomings in the experimental literature. For example, to our knowledge, there are no experimental studies that parallel the WCST administration and a verbal shadowing protocol (for an example of this experimental protocol see^32^). Moreover, there are few studies administrating the WCST to autistic middle adults and there is no study involving old adults. Finally, many studies commonly report an incomplete behavioural profiles computed during WCST administration (e.g. absent NPEs computations). Overall, we have taken in consideration over 39 studies that administrated the WCST to autistic samples (see Table S1 in Supplementary Materials). Among them, we have selected the studies that (a) adopted the Heaton’s version of WCST, (b) involved an autistic group and a matched control group, and (c) reported at least CC, PE, and NPE indices.

The first group^22^ involved 26 children (6 to 12 years) with a diagnosis of autism without mental retardation (DSM-III) and a control group of 52 children matched for age. The second group^23^ involved 13 teenagers ($16.40 \pm 2.84$) with a diagnosis of Asperger syndrome or High-functioning Autism (ICD-10) and a control group of 13 teenagers matched for age and QI. The third group^24^ involves 9 young adults ($27 \pm 7$) with a diagnosis of autism without mental retardation and high verbal competencies (DSM-III) and a control group of 10 young adults matched for age, education and QI. The fourth group^25^ involves 27 middle adults ($33.5 \pm 12$) with a diagnosis of Asperger syndrome (ICD-10) and a control group of 20 middle adults matched for age and QI.

Note that the samples of^24^ and^25^ show similar ages ($27 \pm 7$ vs $37.6 \pm 14.6$), potentially belonging to the same general population. Despite this, we adopt different labels (‘young adults’ and ‘middle adults’) to better distinguish the two groups in this work. We have not found any study administrating WCST to autistic adults over these ages (i.e., ‘old adults’). Lastly, in this work we adopt the term “Autism Spectrum Condition” to refer to the previous diagnostic labels used in these four studies (e.g., Asperger Syndrome, Infantile autism, etc).

Table 1 summaries the demographic information of the eight samples.

**Table 1:** Summary of demographic information about the four selected studies.

	Sample size	Age ($M \pm SD$)	Diagnosis	IQ	Language skills	Comorbidity	Education
**Children**^22^							
ASCs	26	6-12	Infantile Autism (DSM-III)	WAIS: - FSIQ: $80 \pm 12$	$\times$	Absent	Primary school
Controls	42	Matched	NS	$\times$	$\times$	$\times$	Primary school
**Teenagers**^23^							
ASCs	13	$15.60 \pm 3.07$	AS (ICD-10)	WISC-III: - FSIQ: $109 \pm 11.52$	$\times$	$\times$	$\times$
Controls	13	$16.40 \pm 2.84$	NS	WISC-III: - FSIQ: $109.62 \pm 10.36$	$\times$	$\times$	$\times$
**Young adults**^24^							
ASCs	9	$27 \pm 7$	Infantile Autism (DSM-III)	WAIS: - FSIQ: $104 \pm 12$	Absent impairment	$\times$	$12 \pm 2$ years
Controls	10	$28 \pm 5$	NS	WAIS: - FSIQ: $113 \pm 6$	$\times$	Absent	$12 \pm 1$ years
**Middle adults**^25^							
ASCs	27	$37.6 \pm 14.6$	AS (ICD-10)	WAIS-R: - PIQ: $103.7 \pm 19.2$ - VIQ: $106.1 \pm 15.7$	BPVS: $146.5 \pm 13.7$	Absent	$\times$
Controls	20	$33.5 \pm 12$	NS	WAIS-R: - PIQ: $109.4 \pm 18.5$ - VIQ: $107.5 \pm 13.1$	BPVS: $152.6 \pm 7.5$	Absent	Matched

*Matched* not provided information, which however is matched with that of the experimental sample. *Absent* absent comorbidities. $\times$ not provided information, *NS* not significant information (e.g., control groups are neurotypical by definition), *AS* asperger syndrome, *FSIQ* Full Scale Intelligence Quotient, *PIQ* Performance Intelligence Quotient, *VIQ* Verbal Intelligence Quotient.

### Model

The three-component hypothesis of flexible cognition^6,7^ is the theoretical framework at the basis of the computational model. It proposes that flexible goal-directed behaviour is based on the synergy between a self-directed manipulation of representations (e.g., working memory and perceptual representations) and sensory-motor interactions with the external world (e.g., gaze shift and objects displacement). The hypothesis, and the derived computational models, are based on three main components: a hierarchical visual system, a working memory, and a top-down selector of internal representations. We present here a description of the model components and functioning that allows the reader to interpret the presented results. We reported the computational details of the model in Supplementary Materials and a throughout analysis of the model computational architecture and dynamics is presented in^7^. The code of the model is publicly available for online download from the GitHub repository: https://github.com/GiovanniGranato/Flexible-goal-directed-behaviour-and-Inner-Speech.

A first group of components support the internal manipulations of representations. A *hierarchical perceptual component* extracts the input visual features at increasing levels of abstraction (e.g. card features such as colour, shape and size), emulating the human visual system^33^. In the model this component is implemented as a deep generative model. A *working-memory* component stores the ‘priorities’ of the task sub-goals (e.g., the sorting rules) and determines the probability with which the rules are selected. Note that working memory values show a forgetting process caused by a temporal decay, which leads to priority values collapsing to the same baseline. In the brain, this function is mostly supported by the reentrant circuits of frontal cortices^21,34^, and it is implemented here as a recurrent neural network. A *motivational* component exploits the external feedback to update the information in working memory, emulating the ventral basal ganglia^35,36^. It is implemented here as a reinforcement learning (RL) algorithm. A *selector* and a *manipulator* components support the manipulation of perceptual representations. The former chooses the sorting rule to follow on the basis of priorities stored in working-memory. The second implements the manipulation of the internal representations by biasing the perceptual system. For example, in case the selector chooses the colour rule, the manipulator inhibits all the visual features of the card representations with the exception of the ones representing the colour information. The selector and manipulator functions reflect the top-down control that the fronto-parietal cortex and basal ganglia exert on the perceptual cortices^37,38^. In the model these functions are implemented as a softmax function, selecting the rule to follow on the basis of the working memory activations, and a dis-inhibition mechanism, that uses the selected rule to manipulate the high-level representations within the hierarchical generative network. An *inner-speech* component influences the working memory rule selections, on the basis of the selector choices and the external feedback. In particular, this component transmits information on (1) the specific working memory rule whose priority should be changed (e.g., colour), and (2) the positive/negative valence on the basis of external feedback (i.e. the selected rule correctness). Importantly, this component can assume a compensating role with respect to a high working-memory decay and a weak motivational component functioning^7^. Adding a further feedback-dependent bias to the working-memory priorities, this component makes them more distinguishable (e.g., more different priorities values) and more aligned with the task requests. This component is inspired by the brain systems that integrate linguistic and emotional information^39,40^. In the model it is implemented as a deep neural-network that produces an output formed by the valence and the intensity of the required rule change.

The model is also formed by additional components that implement a set of sensorimotor auxiliary functions needed to accomplish the WCST. A *visual sensor* component extracts the visual information from deck cards and target cards. This component is analogous to the eye retina, and in the model it is implemented as an RGB matrix of pixels. A *visual comparator* component executes a visual matching of the deck and selected target card on the base of their manipulated low-level perceptual representations. For example, in case the model chooses the colour rule and the deck, and target cards share the colour feature (e.g., blue), the model produces two blue blobs (coloured blobs are abstract representations of colours^6^) and the visual comparator compares them. In the brain, these processes might rely on an integrated network involving the frontal and temporal-occipital cortices^41,42^. In the model, this function is supported by the computation of the Euclidean distance between the representations of the two cards. A *motor* component controls the saccades on the deck and target cards and moves the deck card close to the chosen target card after a successful visual matching. This component shows a simplified implementation that does not account for potential individual motor differences. This design choice depends on the fact that rule-based category learning is mostly based on a category-based system rather than a procedure system^30^. Indeed, the three-component hypothesis focuses on representation manipulations rather than on motor variability. We have started to consider the representation of learning and sensory-motor differences in autistic population in^31^. At last, after each sorting attempt, a simulated *‘external operator’* component, knowing the correct sorting rule, receives the deck card and the chosen target card and returns positive or negative feedback to the model.

The model has key free parameters that influence its computations and behaviour: ‘error sensitivity’ ($\mu$), representing the magnitude with which the motivational component influences the working-memory sorting-rule priorities in case of negative feedback; ‘memory refresh/forgetting speed’ ($\phi$), representing the decay speed of the working memory rule priorities towards a baseline; ‘distractibility/explorative tendency’ ($\tau$), representing the level of randomness of the rule selection; ‘inner speech contribution’ ($\lambda$), representing the magnitude with which the inner-speech component influences the working memory. The last parameter is the most important for this study. Notably, the number of free parameters is a strength of the model. In fact, by modifying only these four free parameters, the model is able to reproduce many behavioural indices of human samples: there are no other free parameters, and no other changes are made to the model during the simulations.

#### Rationale behind modelling of Inner-Speech

A previous version of the model, without an inner speech component, demonstrated to reproduce the behavioural differences between young adults, old adults, frontal patients, and Parkinson patients in the WCST^6^. An updated version of the model was enhanced with the addition of an inner speech component and validated in^7^. The development, integration, and validation of an inner-speech component was supported both by a theory-driven approach (analysis of literature) and a data-driven approach (experimental validation).

For example, recent studies attested the existence of different kinds of inner speech, such as wilful/deliberative vs. spontaneous inner speech^43^, condensed vs. expanded inner speech, monologic vs. dialogic inner speech, and evaluative/motivational inner speech^1^. Moreover, some authors have highlighted the inner speech relationship with second-order cognition and metacognition^9^.

The inner-speech component of the model exhibits three aspects that contribute to qualifying it as a good emulation of human monological and deliberative / wilful inner-speech. First, this component reinforces the system’s working memory, emulating the phono-articulatory loop in Baddeley’s well-known theory. Second, this component emulates the motivational role of inner-speech for human cognition. Indeed, different authors have underlined the important motivational and self-reinforcing role that inner speech might have (e.g.^10^), even in sport^44^. For example, the questionnaire “Varieties of Inner Speech”^45^ attests that the evaluative/motivating function was the aspect of inner speech more frequently endorsed by participants. Third, the inner-speech component is clearly linked to the definition of ‘second-order cognition’^9^, also called by others ‘metacognition’. Indeed, classical literature considered metacognition mainly as a form of ‘thinking about thinking’^46^. More recently, metacognition has also been investigated in relation to the strategy changes adopted following error detection^47^. Thus, metacognition can be considered as a form of cognitive control, where one sensorimotor system implicitly represents a feature of another system (^48,49^; for a discussion on this, see also^50^). In our model, the inner speech component is strongly intertwined with metacognition. Indeed, the system represents the chosen decision, and evaluates it either positively or negatively, and this influences the strategy that will follow. As shown in^7^, the two processes (the decision on the sorting rule and the evaluation on the operated choice) are closely interwoven, and the sole decision process is not effective in improving the performance.

Overall, our model of inner speech is in line with the way inner speech is intended in the literature. More specifically, it perfectly fits with the kind of deliberative and monologic inner speech.

On the other hand, the model also receives a data-driven validation. Indeed, in^7^ the model reproduced a complete behavioural profile (WCST indices) of three groups of teenagers in different experimental conditions: control, motor tapping, and verbal shadowing (i.e., an experimental protocol disrupting the inner-speech contribution). Importantly, the model parameters that best fit the three samples report an extremely different contribution of the inner-speech to the task solution (a very lower parameter $\lambda$ in verbal shadowing condition). Thus, it demonstrated the ability to disentangle the inner-speech contribution during the WCST. Moreover, in our previous work we have performed many lesions to the inner-speech component. The resulting altered profiles further corroborated the importance of this component (reinforcement of working-memory, motivation and meta-cognition), in line with the theorised role of inner-speech.

Since the model demonstrates these features, we have now exploited it to propose many predictions regarding using inner speech in autistic people. Unfortunately, no experimental manipulation of the inner-speech contribution (e.g., verbal shadowing) is adopted in these experimental works (a literature limitation; see “Limitations and future directions”). Therefore, the different parameters $\lambda$ that our model predicts underpin the samples should be related to individual differences between the control and experimental groups.

Future work should propose new experimental protocols, administrating to autistic samples WCST and a verbal shadowing protocol. This should increase the generalisation of the model (see “Limitations and future directions”).

## Results

### Configurations of parameters of the best fitting models

In^7^ we have used a statistical search method, based on the minimisation of the mean square error (MSE), to find the models’ parameters. In that case, we used this approach to investigate the inner-speech contribution during three experimental conditions (control, motor tapping, verbal shadowing; see “Model”). Here we have adopted the same method. In particular, we have produced many simulations in which we have randomly changed only the four free parameters of the model, keeping the rest of the architecture fixed (see Supplementary Materials for further details). In this way, we have found the parameter configurations that best reproduce the behavioural profile of the control and autistic groups. Although the sample size of some groups is small, the model reproduces the human behavioural data with a low average MSE for both control and autistic groups (see Table S2 in Supplementary Materials).

**Figure 2:**
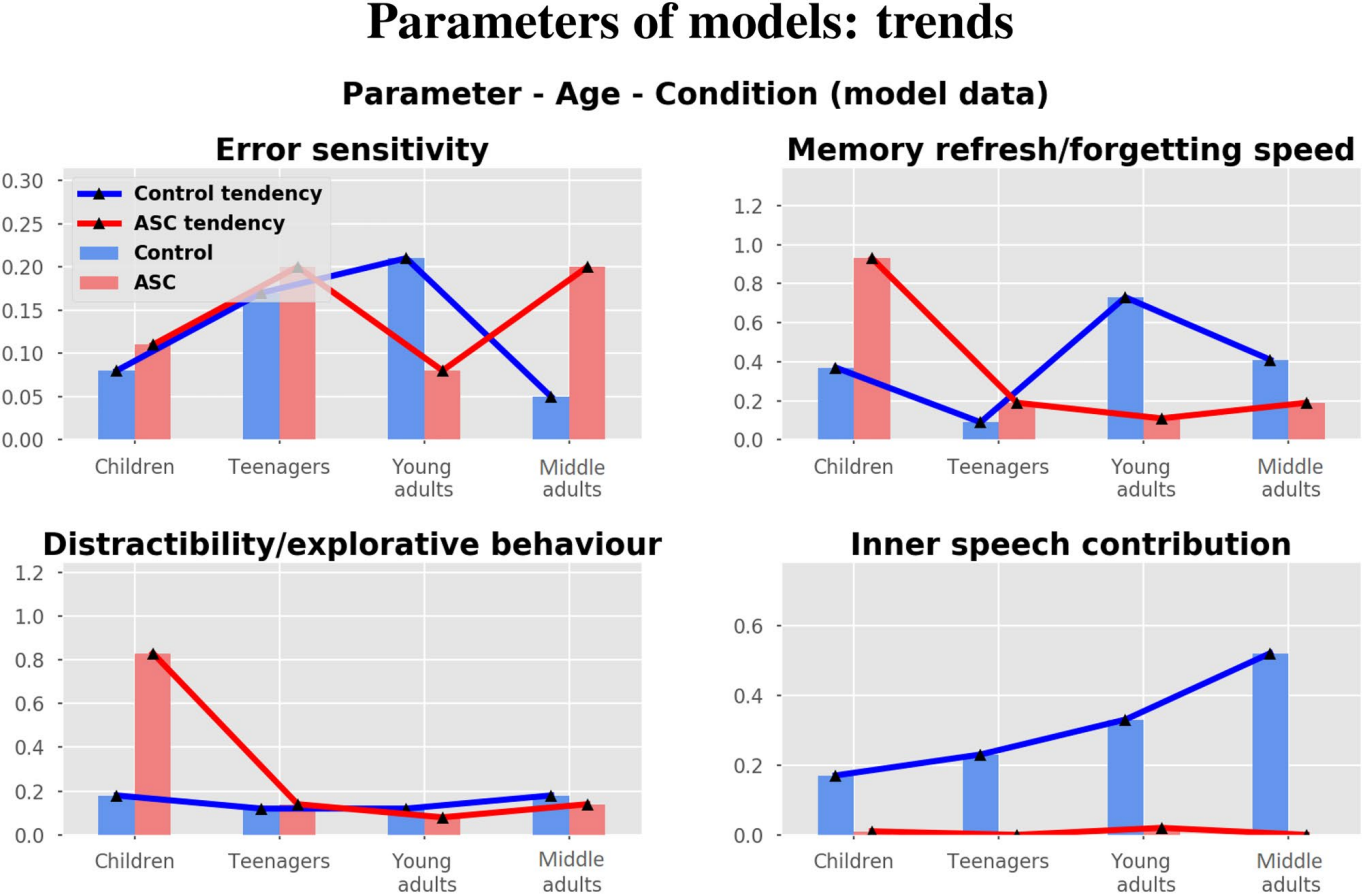
Graphic visualisation of the parameters of the models that best fit the datasets of the human groups (Children, Teenagers, Young adults, Middle adults).

Table 2 and Fig. 2 show the parameter values of the models samples that best fit the dataset of the human groups. The parameters represent the simulated cognitive traits of the model and, therefore, of the modelled human participants.

**Table 2:** Values of the parameters of the models that produce the best fit of the data on the WCST indices.

	Error sensitivity ($\mu$)	Memory refresh, forgetting speed ($\phi$)	Distractibility, explorative behaviour ($\tau$)	Inner speech contribution ($\lambda$)
**Control models**				
Children	0.08	0.37	0.18	0.17
Teenagers	0.17	0.09	0.12	0.23
Young adults	0.21	0.73	0.12	0.33
Middle adults	0.05	0.41	0.18	0.52
**ASC models**				
Children	0.11	0.93	0.83	0.01
Teenagers	0.20	0.19	0.14	0.0
Young adults	0.08	0.11	0.08	0.02
Middle adults	0.20	0.19	0.14	0.0

Regarding the inner speech contribution (parameter $\lambda$), the control groups show an increasing tendency depending on ageing. Differently, ASC groups show an absent or strongly reduced inner speech contribution in all ages.

Regarding the error sensitivity (parameter $\mu$), the control groups show an “inverse U-shaped” curve. In particular, children and middle adults show a similar and lower error sensitivity, while teenagers and young adults show a similar and higher error sensitivity. In the case of ASC groups, there are similarities among pairs of different groups. In particular, children and young adults show a similar and lower error sensitivity, while teenagers and middle adults show the same higher error sensitivity.

Regarding the memory refresh/forgetting speed (parameter $\phi$), the control groups again show similarities between children and middle adults. Differently, teenagers show the lowest value and young adults the highest value. In the case of ASC groups, there is a descending tendency. In particular, children show the highest value with respect to the other groups, which show similar values.

Regarding the distractibility/exploratory behaviour (parameter $\tau$), the control groups have similar values. Despite this, children and middle adults show the same slightly higher value with respect to teenagers and young adults, that show the same value. In the case of ASC groups, similarly to the $\phi$ parameter, we found a descendent tendency. In particular, children show higher value with respect to the other groups, which are similar.

### Behavioural comparisons

Here we introduce many behavioural comparisons between the models that best fit the human groups. Table 3 summarises the behavioural indices while following sections propose detailed statistical analysis.

**Table 3:** Behavioural indices of the models that produce the best fit of the data from participants.

	Completed categories (CC)	Perseverative errors (PE)	Non-preseverative error (NPE)	Failures-to-maintain set (FMS)
**Control models**				
Children	5.06 (0.93)	12.27 (3.26)	14.13 (4.44)	3.06 (1.75)
Teenagers	6.0 (0.0)	10.08 (2.3)	8.62 (3.36)	0.38 (0.62)
Young adults	5.9 (0.3)	6.2 (1.89)	8.5 (4.13)	1.8 (1.72)
Middle adults	5.5 (0.81)	7.9 (2.32)	12.05 (3.53)	2.7 (1.71)
**ASC models**				
Children	0.12 (0.32)	24.77 (4.48)	38.04 (4.4)	0.69 (1.1)
Teenagers	5.08 (1.21)	12.77 (3.12)	13.92 (3.12)	2.85 (1.23)
Young adults	5.44 (0.68)	32.44 (10.23)	14.33 (5.79)	0.11 (0.31)
Middle adults	4.44 (1.03)	12.93 (3.17)	15.07 (4.29)	3.11 (1.59)

#### Comparisons between perseverative errors and non perseverative errors in each group

**Figure 3:**
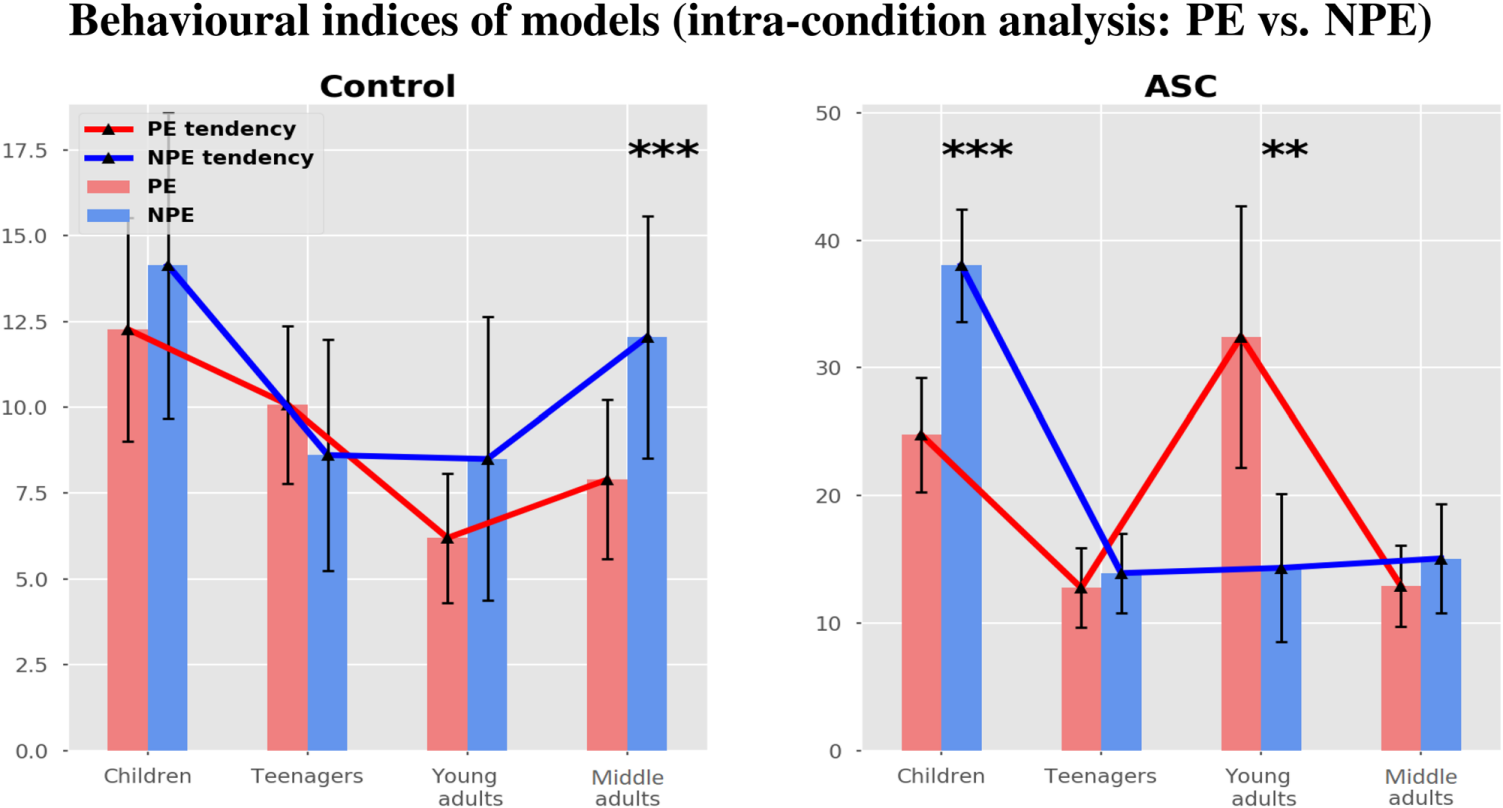
Comparisons between PE and NPE in the control and ASC conditions (Children, Teenagers, Young adults, Middle adults).

Since perseverative errors and non perseverative errors identify two opposite tendencies, respectively for perseveration and for distraction^6^, we performed statistical comparisons (t-tests with Bonferroni’s correction) between PEs and NPEs of each model to investigate its behavioural profile (Fig. 3).

The results show that in the control condition only middle adults show significant differences in their behavioural profile, with an imbalance toward NPE ($7.9 \pm 2.32$ vs $12.05 \pm 3.53$, $p < 0.001$). In the ASC condition, children show an imbalance toward NPE ($24.77 \pm 4.48$ vs $38.04 \pm 4.4$, $p < 0.001$) while young adults show an imbalance toward PE ($32.44 \pm 10.23$ vs $14.33 \pm 5.79$, $p < 0.01$). Although the plots show many PEs/NPEs imbalances in the other models, high sample variability prevents further statistical differences.

#### Comparison between the behaviour of different age groups (intra-condition analysis)

**Figure 4:**
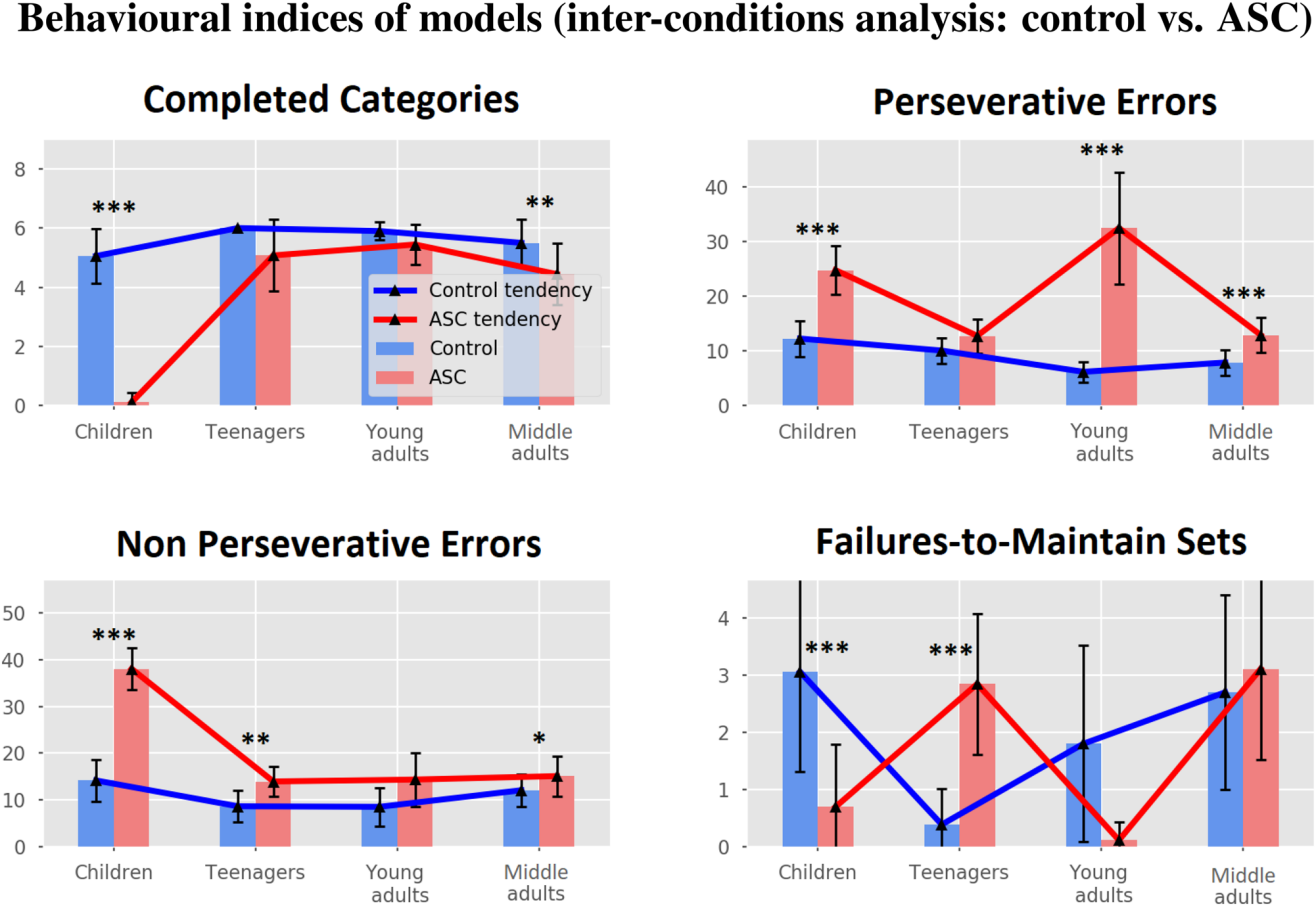
Behavioural indices and comparisons of all models (Children, Teenagers, Young adults, Middle adults).

We have performed statistical comparisons (one-way Anova and post-hoc t-tests with Bonferroni’s correction) between the models of each condition (Fig. 4, blue and red trend lines).

Regarding completed categories index (CC), there are statistical difference between the control models ($F = 7.03$, $p < 0.001$). Post hoc tests (Table S3 in Supplementary materials) indicate that children achieve a lower CC index with respect to teenagers ($5.06 \pm 0.93$ vs $6.0 \pm 0.0$, $p < 0.001$). There are no significant statistical differences between the other models, possibly due to the high variability of each model sample. We found statistical difference between the ASC models ($F > 50$, $p < 0.001$). Post hoc tests (Table S4 in Supplementary Materials) indicate that children achieve a low CC index with respect to teenagers ($0.12 \pm 0.32$ vs $5.08 \pm 1.21$, $p < 0.001$), young adults ($0.12 \pm 0.32$ vs $5.44 \pm 0.68$, $p < 0.001$), and middle adults ($0.12 \pm 0.32$ vs $4.44 \pm 1.03$, $p < 0.001$). We did not find further significant statistical differences between the other models.

Regarding perseverative errors (PE), there are statistical differences between the control models ($F = 19.87$, $p < 0.001$). Post hoc tests (Table S5 in Supplementary Materials) indicate that children have higher PE with respect to young adults ($12.27 \pm 3.26$ vs $6.2 \pm 1.89$, $p < 0.001$) and middle adults ($12.27 \pm 3.26$ vs $7.9 \pm 2.32$, $p < 0.001$). There are no further significant statistical differences between the other models. There are statistical differences between ASC models ($F > 50$, $p < 0.001$). Post hoc tests of ASC models (Table S6 in Supplementary Materials) indicate that children show higher PE with respect to teenagers ($24.77 \pm 4.48$ vs $12.77 \pm 3.12$, $p < 0.001$) and middle adults ($24.77 \pm 4.48$ vs $12.93 \pm 3.17$, $p < 0.001$), and lower PE with respect to young adults ($24.77 \pm 4.48$ vs $32.44 \pm 10.23$, $p < 0.001$). There are no further significant statistical differences between teenagers and young adults.

Regarding non perseverative errors (NPE), there are statistical difference between control models ($F = 9.82$, $p < 0.001$). Post hoc tests (Table S7 in Supplementary Materials) indicate that children have higher NPE with respect to teenagers ($14.13 \pm 4.44$ vs $8.62 \pm 3.36$, $p < .001$) and young adults ($14.13 \pm 4.44$ vs $8.5 \pm 4.13$, $p < 0.01$). there are no further significant statistical differences between the other models. There are statistical differences between ASC models ($F > 50$, $p < 0.001$). Post hoc tests of ASC models (Table S8 in Supplementary Materials) indicate that children have higher NPE with respect to teenagers ($38.04 \pm 4.4$ vs $13.92 \pm 3.12$, $p < 0.001$), young adults ($38.04 \pm 4.4$ vs $14.33 \pm 5.79$, $p < 0.001$), and middle adults ($38.04 \pm 4.4$ vs $15.07 \pm 4.29$, $p < 0.001$). there are no further significant statistical differences between the other models.

Regarding failure-to-maintain sets errors (FMS), there are statistical differences between control models ($F = 10.04$, $p < 0.001$). Post hoc tests (Table S9 in Supplementary Materials) indicate that children have higher FMS with respect to teenagers ($3.06 \pm 1.75$ vs $0.38 \pm 0.62$, $p < 0.001$), and middle adults have higher FMS with respect to teenagers ($2.7 \pm 1.71$ vs $0.38 \pm 0.62$, $p < 0.01$). There are no further significant statistical differences between the other models. There are statistical differences between ASC models ($F = 24.31$, $p < 0.001$). Post hoc tests of ASC models (Table S10 in Supplementary Materials) indicate that children have lower FMS with respect to teenagers ($0.69 \pm 1.1$ vs $2.85 \pm 1.23$, $p < 0.001$) and middle adults ($0.69 \pm 1.1$ vs $3.11 \pm 1.59$, $p < 0.001$). Moreover, teenagers have higher FMS with respect to young adults ($2.85 \pm 1.23$ vs $0.11 \pm 0.31$, $p < 0.001$), and young adults have lower FMS with respect to middle adults ($0.11 \pm 0.31$ vs $3.11 \pm 1.59$, $p < 0.001$). There are no further significant statistical differences between the other models.

#### Comparison between the behaviour of the control and experimental groups (inter-condition analysis)

We have performed statistical comparisons (t-tests with Bonferroni’s correction) between the indices of the control and ASC models to investigate the behavioural differences between them in each age (Fig. 4).

Regarding completed categories (CC), they are lower in ASC children ($5.06 \pm 0.93$ vs $0.12 \pm 0.32$, $p < 0.001$) and ASC older adults ($5.5 \pm 0.81$ vs $4.44 \pm 1.03$, $p < 0.01$). We did not find a statistical differences in teenagers ($6.0 \pm 0.0$ vs $5.08 \pm 1.21$, $p > 0.05$) and young adults ($5.9 \pm 0.3$ vs $5.44 \pm 0.68$, $p > 0.05$).

Regarding perseverative errors (PE), they are higher in ASC children ($12.27 \pm 3.26$ vs $24.77 \pm 4.48$, $p < .001$), ASC young adults ($6.2 \pm 1.89$ vs $32.44 \pm 10.23$, $p < 0.001$), and ASC middle adults ($7.9 \pm 2.32$ vs $12.93 \pm 3.17$, $p < 0.001$). There is no statistical difference in teenagers ($10.08 \pm 2.3$ vs $12.77 \pm 3.12$, $p > .05$).

Regarding non perseverative errors (NPE), they are higher in ASC children ($14.13 \pm 4.44$ vs $38.04 \pm 4.4$, $p < 0.001$) and in ASC teenagers ($8.62 \pm 3.36$ vs $13.92 \pm 3.12$, $p < 0.01$). There is no statistical difference in young adults ($8.5 \pm 4.13$ vs $14.33 \pm 5.79$, $p > 0.05$) but there is a slightly higher value in ASC middle adults ($12.05 \pm 3.53$ vs $15.07 \pm 4.29$, $p < 0.05$).

Regarding failure-to-maintain set errors (FMS), they are lower in ASC children ($3.06 \pm 1.75$ vs $0.69 \pm 1.1$, $p < 0.001$) and higher in ASC teenagers ($0.38 \pm 0.62$ vs $2.85 \pm 1.23$, $p < 0.001$). There are not statistical difference in young adults ($1.85 \pm 1.72$ vs $0.11 \pm 0.31$, $p > 0.05$) and middle adults ($2.7 \pm 1.71$ vs $3.11 \pm 1.59$, $p > 0.05$).

### Internal functioning comparisons

**Figure 5:**
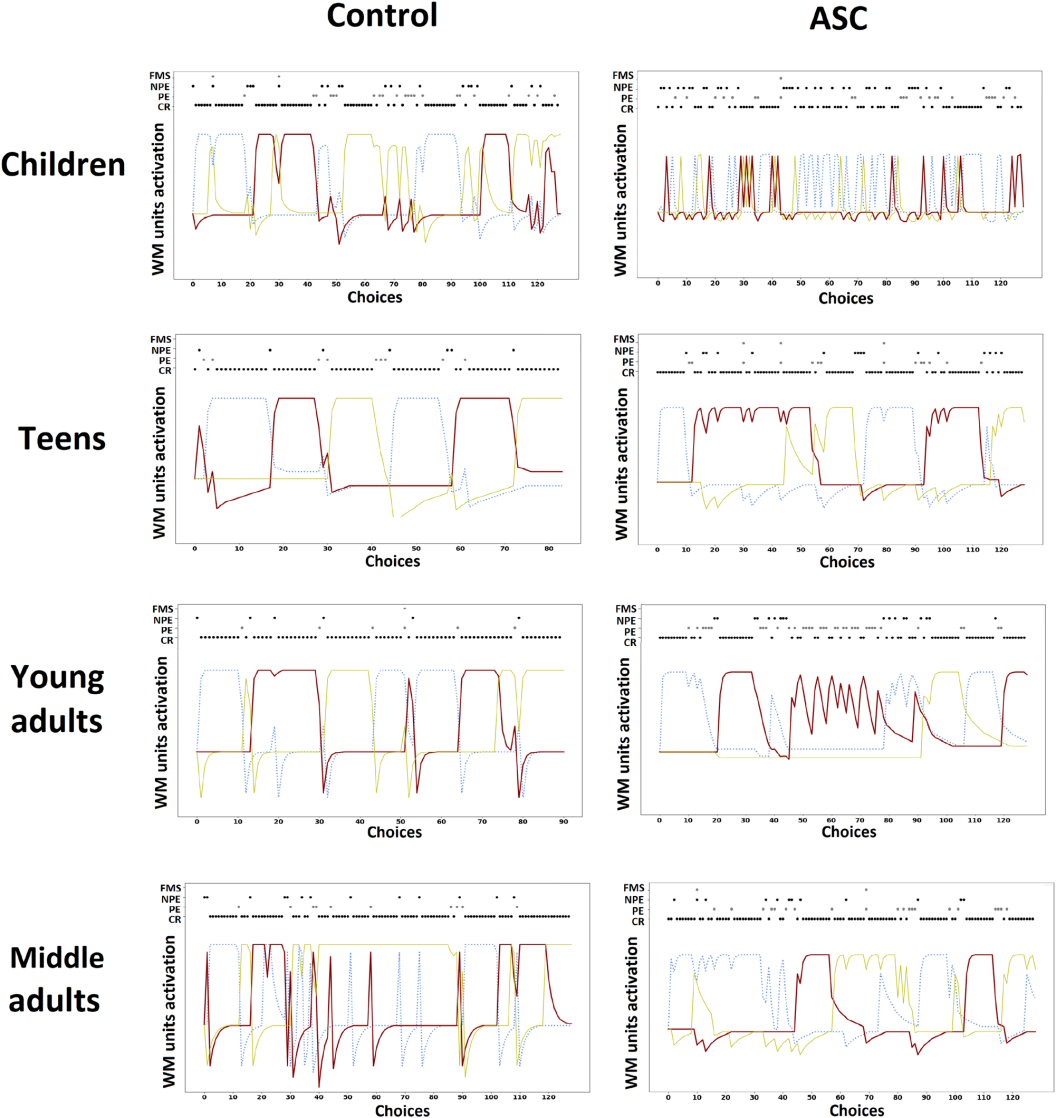
Internal functioning of the executive working memory of the control and ASC models. Each line represents the activation of a memory unit encoding a specific matching rule: thick red line: colour-based matching rule; dotted thin blue line: shape-based matching rule; continuous yellow line: size-based matching rule. The dots at the top of graphs indicate the instances of correct responses (CR) or errors (PE, NPE, FMS).

We have also investigated the internal functioning of the models. Figure 5 shows the internal activation of the working memory units of the models, recorded during their task performance. The activation of each unit corresponds to a specific sorting rule to follow, and the top space of each plot of the figure shows the errors that occur during each card response.

In the case of children, the control and ASC models appear very different. In particular, the ASC model shows several erratic strategy changes that cause the occurrence of several NPEs. Interestingly, even though the model is evidently distracted and does not keep the focus on a specific strategy, some PEs are still scored. As we have already shown in^7^, a participant with high distractability can choose by chance an already tried strategy, thus erroneously appearing perseverative. Here we refer to these errors as ‘distraction-related PEs’. At last, also the control model shows a sub-optimal performance caused by reasoning errors (e.g., see the 65–80 interval of trials) and attention failures (e.g. 3–4 interval and 25–35 interval).

In the case of teenagers, the control model shows a good landscape with some negligible reasoning failures (e.g., 0–5 interval) and perseveration (e.g., 40–45 interval). The ASC model shows several ‘sustained attentional failures’ (e.g., 10–40 interval) and reasoning errors (e.g. 110–120 interval) that cause many NPEs and FMSs errors.

In the case of young adults, the control model shows a good landscape with minor attention failures (e.g., 15–20, 50–55 intervals). The ASC model shows many perseverative behaviours (e.g., 40–70 interval) and attention failures (e.g., 75–85 interval).

In the case of middle adults, both control and ASC models show sub-optimal landscapes. The ASC model shows many attention failures (e.g., 35–45 interval) and reasoning errors (e.g., 55–65 interval), causing many PEs and NPEs. Interestingly, the control model shows as many FMSs (e.g., 50–75 interval) as the ASC model, showing poorly focused behaviour.

## Discussion

The computational model we have presented here reproduces most behavioural indices of control of autistic humans groups performing the Wisconsin Cards Sorting Test. Moreover, it suggest s several intra-group and inter-group cognitive and behavioural differences.

Regarding control samples, we generally have found similar parameters values between children and middle adults (Fig. 2, blue lines) in error sensitivity, memory refresh/forgetting speed, and distractibility/exploratory behaviour, detecting some ‘U-shaped tendencies’ related to age. Differently from the other three parameters, we found an inner-speech contribution that increases with age, being low in children and high in adults. Further investigations of the cognitive profile of control groups confirmed the U-shaped trends in perseverative errors (perseverative behaviour) and non-perseverative errors (attention/reasoning failures) (Fig. 3, left plot). At last, a qualitative analysis of internal activations of the models corroborated these trends (Fig. 5), showing that teenagers and young adults perform better than children and middle adults who exhibited more distracted and perseverative behaviours.

Despite the emergence of these trends, the cognitive differences (parameters) between controls groups do not always cause statistically significant differences in behavioural data (Fig. 4). For example, only children show significantly lower global performances than the other groups (teenagers, young adults, and middle adults), which do not show statistical differences.

These results allow the interpretation of contrasting findings on ageing-related effects. In particular, several studies indicate that ageing causes significant brain changes (e.g.^51,52^) and a weakening of executive functions^53–55^. However, other studies reveal compensatory brain processes such as functional reorganisation and increased bilateral recruitment^56,57^.

Considering this literature and the dynamics of our model, we propose here an interpretation of these results. The inner speech contribution, showing an increasing trend from children to middle adults, could play an ageing compensation effect. It could *support early development* and *avoid/compensate cognitive decline*, thus mitigating the life-span cognitive and behavioural differences between neurotypical individuals. As we demonstrated in^7^, inner speech can interact with other cognitive processes, compensating for specific deficits (e.g., weak working memory storage capacity or weak feedback processing). In particular, it can decrease distracted and perseverative behaviours, thus boosting global performance. Our proposal agrees with several studies highlighting that inner speech could provide developmental support for immature executive functions in children^1^ and executive compensative/modulatory function in middle adults (e.g.^26,58,59^).

Regarding autistic sample, the model predicts relevant differences in cognitive profiles with respect to control samples. First, autistic groups show a reduced contribution of inner speech along the life-span. Second, there are greater differences between children and other groups regarding working memory decay and distractibility. Third, autistic groups show different imbalances with respect to control groups (Fig. 3, right plot). In particular, autistic children show an evident imbalance toward distractibility (NPEs), while young adults show an imbalance toward perseverative behaviours (PEs).

These results are particularly interesting because the diagnostic criteria for autism rely on repetitive behaviours^12^ and clinical studies mostly focus on perseverative/repetitive behaviours in autistic children and adults^60,61^. On the other side, several works have investigated attention abnormalities in autism, suggesting that an attention impairment could play a causal role in the development of autistic individuals (for a review, see^62^). The results presented here agree with these last studies, suggesting that autistic children mostly show an imbalance toward distractions with respect to perseverative behaviours. Moreover, the model suggests a cognitive change in autistic people along the life span, from a distracted profile in children to a perseverative one in young adults.

Regarding age-related behavioural differences, the cognitive traits (parameters) seem to have a more marked effect on behaviours in autistic peoples than in the control groups. For example, the descending values of distractibility and memory refresh are reflected by a similar curve of NPEs, and the low error sensitivity in children and young adults cause higher PEs with respect to teenagers and middle adults. However, in the case of children, this result is evidently altered by many distractibility-related PEs^7^. In particular, the extreme distractibility of autistic children causes a random behaviour (Fig. 5, first row) that is sometimes scored as ‘perseverative behaviour’ even if attention failures cause it (see the imbalance toward NPEs in Fig. 3, right plot).

Interestingly, the FMS curve shows a different and unexpected trend with respect to the control trends. In particular, we might expect autistic children to show higher FMS due to high distraction; however, they show a low value of this index. This is probably explained by the difference between NPEs and FMS, where the first indicate an attentional/reasoning failure and the second indicate a sustained attention failure.

Since autistic children cannot focus on a specific strategy (e.g., a sorting rule) for long^63^, they often do not achieve the necessary number of responses to occur in a sustained attention error (e.g., FMS). These results are coherent with^64^, detecting impairment in selective attention and not sustained attention, and with^65^, detecting a response inhibition impairment rather than a sustained attention impairment. A high FMS in middle adults is another interesting result. While a higher FMS value for teenagers is expected and corroborates a sustained attention deficit^66,67^, we could expect an imbalance toward perseveration in middle adults. Instead, we have found higher FMS with respect to young adults without a marked PEs/NPEs imbalance (Fig. 3, right plot). These results need further investigations, in particular regarding sustained attention in autistic middle adults.

In summary, comparing control and autistic samples, we have found statistically lower performance only in autistic children and autistic middle adults with respect to their control groups. The behaviour comparisons (Fig. 4) and the analysis of the internal activation of the models (Fig. 5) suggest that these differences are caused mainly by more distractions in autistic children/teenagers and a higher perseveration in autistic young/middle adults. In^6^ we have highlighted that PEs are mostly caused by alterations of sub-cortical structures and frontal medial cortices. On the other hand, an alteration of dorsal frontal cortices correlates with many types of errors, among which NPEs. Since brain maturation follows a sub-cortical/cortical progression^68,69^, we should expect this behavioural progression from NPEs to PEs. However, the presence of a more marked progression in autistic samples could depend on an atypical brain maturation process^67,70,71^. Unfortunately, a marked imbalance toward PEs in young adults remains even after a complete brain maturation. Consistent with the experimental evidence^31,72,73^, this could depend on a residual imbalanced effect of the sub-cortical structures on cortical systems.

Overall, our results corroborate the idea of immature executive functioning in autistic children and slight cognitive decline in middle adults, as suggested by similar trends in the control groups. Interestingly, control groups show weaker intra-condition behavioural differences than autistic groups, where the difference between age groups appears more marked. Although many latent variables can contribute to these age-dependent difference in behavioural performances (e.g., an altered ageing process speculated above or an impaired social learning in autistic children, see^74^ for a review), we propose here that a reduced inner speech development in autistic people could make the ageing effects more evident. In particular, since in control conditions the inner speech represents cognitive support, its reduction in autistic people could deprive them of these compensation processes.

Our hypothesis can contribute to explaining the contrasting evidence of studies on autism, inner speech, and executive functions (for a review, see^14^). In particular, the differences might be caused by heterogeneous samples involved, spanning from children to middle adults. Moreover, this proposal is coherent with many studies regarding autism and life-span cognitive changes^75,76^, suggesting that also autistic people show an improvement in executive functions during the life-span. Indeed in autistic teenagers and young adults, compensating processes could emerge (e.g., higher visual skills and visual thinking with respect to neurotypical peoples^77–79^). However, the reduction of inner-speech support could still represent a substantial impairment for children and middle adults.

Finally, while this work considers an experimental setting and hence has limited ecological validity^28^ , our results have interesting clinical implications. Many therapeutic approaches aim to limit compromising symptoms in autism^80^, but only a few of them focus on speech abilities in autism^81–83^. These approaches aim to increase linguistic skills to improve social communication abilities, but they do not directly focus on self-directed language (inner-speech). This study suggests that clinicians should design a new class of therapeutic approaches primarily focusing on developing inner speech skills in autistic children. In particular, the integration of early development of inner speech and strong visual thinking could represent important cognitive support throughout the life span of autistic people, from childhood to adulthood.

### Limitations and future directions

This work successfully involves participatory research, as the first author of this study is autistic and a researcher of the laboratory^84^. Moreover, it integrates computational modelling approaches for clinical scopes. Despite these strengths, this work shows limitations that we aim to overcome in our future works.

First, the experimental studies we have considered^22–25^ do not include a verbal shadowing protocol that directly evaluates the inner-speech contribution. Despite this, our model has already demonstrated to disentangle the inner-speech contribution during an experimental protocol that integrates the WCST with verbal shadowing protocol^7^. Here we have exploited the model to propose inferences about individual differences, and the predictions are compatible with the proposals of the literature, also producing useful interpretations for clinicians. Future experimental studies with autistic people should integrate the administration of WCST with verbal shadowing as done in^32^. We will consider future experimental data with the aim of testing the model’s predictions and building a comprehensive theory of experimental, neuropsychological, and computational aspects of Autism.

Second, the sample size of groups extracted from^23^ and^24^ is small. Although we have detected inter-groups statistical differences (e.g., more PEs in autistic young adults or more NPEs in autistic teenagers), this factor could lead to no difference in global performances in these groups. Future experimental studies aim to enlarge the sample size of experimental groups, making the interpretations regarding inner-speech and autism more robust.

Third, the age difference between young adults ($27 \pm 7$) and middle adults ($37.6 \pm 14.6$) is not large, and it could alter our results. This point represents a general lack of literature on autistic people, in fact we have not found studies administrating the WCST to autistic adults older than such age. New experimental studies should aim to cover a wider age range, especially among autistic middle adults. This could get clinicians to consider Autism as a life-span condition, from childhood to old age.

Finally, we have used our model to explain the behavioural data of four different age groups. Interestingly, our model predicts several age-related cognitive and behavioural trends. Despite these meaningful results, we could not test our model with other groups (see “Task and participants data”). Therefore, our age-related findings should be validated by increasing the number of groups for each age. We aim to test the generalizability of our findings with further experimental works in the future, perhaps by prompting experimental and computational collaborations.

## Conclusions

This study investigates the relationship between inner speech, ageing, and autism spectrum condition. The study uses a previously validated computational model of the Wisconsin sorting card test to reproduce and interpret the data of eight groups of participants differing in age and condition (control condition vs. autism spectrum condition). The model predicts that in the control condition, inner speech contribution increases from childhood to adulthood, possibly supporting early development in children and compensating for cognitive decline in middle adults. Conversely, the results suggest that autistic people exhibit a reduced inner speech contribution without differences between different ages. Moreover, autistic children and middle adults have shown lower performances than their matched controls. Hence, we propose here reduced inner speech support in autistic people could create difficulties in early development and life-span ageing effects. This hypothesis has clinical implications, suggesting that psycho-therapeutic approaches should focus more on developing inner speech skills in autism spectrum conditions.

## Supplementary Information

Supplementary Information.

## Data Availability

All data generated or analysed during this study are included in this published article and its supplementary information files.
