# Supplementary material for: A computational model of inner speech supporting flexible goal-directed behaviour in Autism
Source: Sci Rep. 2022 Aug 20;12:14198. doi: 10.1038/s41598-022-18445-9 (PMC9392752; doi:10.1038/s41598-022-18445-9)
Supplement: Supplementary file 1 — Supplementary Information. [file 41598_2022_18445_MOESM1_ESM.pdf]

A COMPUTATIONAL MODEL OF INNER SPEECH SUPPORTING FLEXIBLE GOAL-DIRECTED BEHAVIOUR IN AUTISM
Supplementary Materials

Giovanni Granato*
Laboratory of Computational Embodied Neuroscience
Institute of Cognitive Sciences and Technologies
National Research Council of Italy,
Rome, Italy
School of Computing, Electronics and Mathematics
University of Plymouth
Plymouth, U.K.
giovanni.granato@istc.cnr.it

Anna M. Borghi
Department of Dynamic and Clinical Psychology
Sapienza University of Rome
Institute of Cognitive Sciences and Technologies
National Research Council of Italy
Rome, Italy
anna.borghi@uniroma1.it

Andrea Mattera
Laboratory of Computational Embodied Neuroscience
Institute of Cognitive Sciences and Technologies
National Research Council of Italy
Rome, Italy
andrea.mattera@istc.cnr.it

Gianluca Baldassarre
Laboratory of Computational Embodied Neuroscience
Institute of Cognitive Sciences and Technologies
National Research Council of Italy
Rome, Italy
gianluca.baldassarre@istc.cnr.it

August 11, 2022

1 Review process underlying our selection of four experimental studies

The selection of four specific experimental studies in this work is the product of an extended literature review process. In particular, we have taken in consideration all studies that administrate the WSCT to autistic samples. Then, we used three criteria for selecting those we used here: (a) they adopted the Heaton's version of WCST, (b) they involved an autistic group and a matched control group, and (c) they reported at least CC, PE, and NPE indices.

Table S1 reports the 39 studies we initially considered, of which the last four are the ones we selected for this modelling work.

	**Heaton's WCST (Criterion A)**	**Control sample and ASC sample (Criterion B)**	**Complete WCST profile (Criterion C)**
**Considered studies**			
[1]	✗	✓	✗
[2]	✓	✓	✗
[3]	✓	✓	✗
[4]	✓	✓	✗
[5]	✓	✓	✗
[6]	✓	✓	✗
[7]	✓	✓	✗
[8]	✓	✓	✗
[9]	✓	✓	✗
[10]	✓	✓	✗
[11]	✓	✓	✗
[12]	✓	✓	✗
[13]	✓	✓	✗
[14]	✓	✓	✗
[15]	✓	✓	✗
[16]	✓	✓	✗
[17]	✗	✓	✗
[18]	✗	✓	✗
[19]	✓	✓	✗
[20]	✗	✓	✗
[21]	✗	✓	✗
[22]	✓	✓	✗
[23]	✓	✓	✗
[24]	✗	✗	✗
[25]	✓	✓	✗
[26]	✗	✓	✗
[27]	✓	✗	✗
[28]	✓	✗	✗
[29]	✓	✓	✗
[30]	✗	✓	✗
[31]	✗	✗	✗
[32]	✗	✗	✓
[33]	✓	✓	✗
[34]	✓	✓	✗
[35]	✗	✗	✗
**Selected studies**			
[36]	✓	✓	✓
[37]	✓	✓	✓
[38]	✓	✓	✓
[39]	✓	✓	✓

Table S1: Studies we considered during our selection process.

2 Computational details of the model

Environment The cards we used are polygons with a unique combination of three visual dimensions (colour, form, and size), each having one of four possible attributes: colour (red, green, blue, yellow); form (square, circle, triangle, bar); size (large, medium-large, medium-small, small). There are thus $4^3 = 64$ combinations (cards) of attributes. We created a simulated environment composed by the objects (cards) which the model can visually explore (visual search) and on which it can executes a physical action (displacement).

Visual sensor The visual sensor returns a $28 \times 28 \times 3$ RGBY pixel matrix, representing a limited portion of the whole virtual table. The visual sensor is actively moved, in a top-down way (visual search), toward the deck and then sequentially toward the target cards. These matrices are then flattened in a vector of 2352 elements and represent the perceptual input to the model.

Working-memory The working-memory is formed by three recurrent units, each having a self-connection, which can acquire a continuous value ranging in $[0, 1]$. The activation of the each unit is characterised by an internal decay toward a baseline (0.5) and is described by the following equation:

$$m_{l,t} = (1 - \phi) \cdot m_{l,t-1} + \phi \cdot \alpha = m_{l,t-1} + \phi(-m_{l,t-1} + \alpha) \tag{1}$$

where $m_{l,t}$ is the value related to a losing unit l ($l \in 1, 2, 3$; $l \neq s$, where s is the selected unit considered below) at time t, $1 - \phi$ is the strength of the recurrent connection, and $\alpha = 0.5$ is the baseline value to which the memory unit activation converges. The activation of each unit represents the likelihood of selection that the system assigns to each of the three possible matching rules of the task related to colour, form, and size. The parameters ϕ is a critical parameter of the model investigated in the simulations.

Motivational component This component is supported by a reinforcement learning algorithm. In particular it receives the external feedback signal (a binary value in $\{0, 1\}$) and subsequently affects the activation of the unit encoding the last selected and used rule, as follows:

$$m_{s,t} = (1 - \mu) \cdot m_{s,t-1} + \mu \cdot r = m_{s,t-1} + \mu(-m_{s,t-1} + r) \tag{2}$$

where $m_{s,t}$ is the new activation of the rule unit, $s \in \{1, 2, 3\}$ is the index of the selected rule, $m_{s,t-1}$ is the current activation of the unit, $(1 - \mu)$ is the strength of the unit recurrent connection, μ regulates the impact of the feedback on the memory, and r is the feedback signal that is equal to 1 in case of positive feedback (correct matching of the deck card and target card) and 0 otherwise. The parameter μ is set to a fixed value of 0.7 for positive feedback and to a variable value for the negative feedback. The latter value is a critical parameter of the model investigated in the simulations.

Hierarchical perceptual component This component is supported by a deep generative model, in particular a *Deep Belief Network* (DBN, [40]) composed of two stacked *Restricted Boltzmann Machines* (RBM). We trained the first RBM, composed of the input layer and the first hidden layer of DBN, with a classical unsupervised learning algorithm for this model (*contrasting divergence*, [41]). We trained the second RBM, composed by the first and second hidden layers of the DBN, with a modified version of the original algorithm that allows us to alter the reconstructions of original inputs to obtain prototypical representations of input image features on which the system focuses on (e.g., in case of a focus on colour, a red triangle given as input is reconstructed as a shapeless red blob). This modification causes the emergence of three groups of units in the last layer of DBN (its second hidden layer), each corresponding to specific visual categories of the input (first four units for colour: red, green, blue, yellow; second four units: square, circle, bar, triangle; third four units: small, medium-small, medium-large, large). The model is able to 'reconstruct' ('generate') the original input trough a bidirectional activation from the input layer, to the hidden layer, and then back to the input layer. In particular, the selector and manipulator considered below are able to select one category (one group of four units), and one attribute within it (one neural unit), to produce the prototypical rule-based reconstruction of images mentioned above.

Selector and manipulator components The selector is supported by a $softmax$ function, a winner-take-all (WTA) function that receives the values from the working memory as input, and chooses the matching rule as follows:

$$Pr(k = s) = \frac{\exp(m_k \,/\, \tau)}{\sum_{q=1}^{3} \exp(m_q \,/\, \tau)} \tag{3}$$

where $Pr(k = s)$ is the probability that the matching rule k ($k \in 1, 2, 3$) is selected ($k = s$). The parameter τ of the $softmax$ function, called 'temperature', regulates the randomness of the selection and is the third important

parameter manipulated in the simulations. A high value of τ causes a high randomness/exploration of the decisions. The probabilities $Pr(\cdot)$, summing up to 1, are used to stochastically select the matching rule to use. The manipulator is composed by two layers of 3 units, linked with one by one negative projections. Each unit of the second layer is always active and has negative projections to a specific group of the last layer of the perceptual component, so the activation of a specific unit in the first layer of the manipulator causes a disinhibition of the corresponding group of the last layer of the perceptual component. Moreover, the manipulator implements a *Hard-max* function leading to select only one unit (attribute) within the each group (category) of four units.

Verbal component This component is supported by a multi-layer perceptron (MLP), formed by 4 input units, 10 sigmoid hidden units, and 3 output linear units. In particular it receives one-to-one connections from the selector units and sends one-to-one connections to the WM units. This process is in particular implemented as follows:

$$m_t = m_{t-1} + \lambda \cdot L_t \tag{4}$$

where m_t is the new activation of a WM rule unit, m_{t-1} is the current activation of the WM unit, λ represents the strengths of the one-to-one connection weights linking the language component output-layer units to the WM units, L_t is the current activation of the language component output layer caused by the previous selector units' activation (this time mismatch implies that the component implements a phonological memory).

The input-layer is formed by 4 units, i.e. the selector winner-takes-all *one-hot vector* activation and the binary incorrect/correct match feedback encoded with respectively 0/1. We trained the MLP to activate the output-layer 3 units as follow: the unit corresponding to the selected rule learned to produce a $-1/+1$ value based on the match/mismatch feedback; the other two units activated with 0. The language component is activated two times to simulate: (a) the phonological-loop working memory; (b) the feedback-dependent verbal update of the main working memory. In the first activation, the component input layer is activated by the one-hot code of the selector while its feedback unit is activated with 1 (meaning 'maintenance of the current rule'). In the second activation, the component input layer is activated by the selector activation, but in this case the feedback unit value is activated on the basis of the external feedback (0/1), obtained after the action execution (displacement of the card). The contribution of language to the working memory is regulated by a coefficient λ that ranges in $[0, 1]$ and represents the strengths of the one-to-one connection weights linking the language component output layer to the main working memory units. The coefficient λ is the fourth and last important parameter regulating the functioning of the model and investigated in the simulations. The language MLP component is trained before the experiments illustrated in the main text with the backpropagation (supervised learning)

Visual comparator This component is supported by a function that computates the Euclidean distance between the two reconstructed images corresponding to the deck card and the currently-foveated target card returned by the perceptual component. It returns a Boolean value representing the result of the comparison ('same'/'not same').

Motor component This component allows (a) the top-down visual search, i.e. the saccades corresponding to the displacement of visuals sensor, and (b) the interaction of the model with environment (displacement of the deck card from the deck to specific target card). The first mechanism receives the position (Cartesian coordinates) of the deck card and the target cards and displaces the visual sensor on them in a sequential manner. The second mechanism receives the position of the deck card and of the matched target card (Cartesian coordinates) and displaces the deck card toward the position of the matched target card.

3 Results

3.1 Fitting results and comparison between the behaviour of the models and of human groups: models validation details

We adopted the same procedure corroborated in [42] to execute the parameters search, aimed to find the parameters of the models that produce the behaviour that best fits those of human populations. In particular we randomly-sampled $3,000$ combinations of parameters each drawn with a uniform distribution in the following ranges: ϕ: $(0.0, 1.0)$; μ: $(0.0, 1.0)$; τ: $(0.0, 0.3)$; λ: $(0.0, 1.0)$. For each parameter combination, we then performed 30 simulations of the task, so obtaining an average value of the WCST indices. We finally computed the Minimum Squared Errors (MSEs) between the WCST indices of the models and human population, as follows:

$$MSE = \frac{||\mathbf{y} - \mathbf{y}'||_2^2}{n} \tag{5}$$

where $\mathbf{y}$ is the vector of mean indices of the human group, $\mathbf{y}'$ is the vector of mean indices of the considered parameter combination, $|| \cdot ||_2^2$ is the square of the L2 norm, and n is the length of vectors.

Table S2 shows the MSEs for each experimental group while following plots show the comparisons (t-tests) between the human groups and the model groups for each index. Mostly indices are not statistically different, suggesting that the behaviour of the eight models fits the corresponding human populations.

Minimum Squared Errors (MSEs)

	Control	ASC	Means
**Children**	$1.2 * 10^{-4}$	$6.9 * 10^{-5}$	$9.5 * 10^{-5}$
**Teenagers**	$4.0 * 10^{-5}$	$2.5 * 10^{-5}$	$3.25 * 10^{-5}$
**Young adults**	$4.4 * 10^{-5}$	$1.2 * 10^{-4}$	$8.2 * 10^{-5}$
**Middle adults**	$0.70 * 10^{-5}$	$3.5 * 10^{-5}$	$2.1 * 10^{-5}$
**Means**	$5.3 * 10^{-5}$	$6.2 * 10^{-5}$	$5.8 * 10^{-5}$

Table S2: Minimum Squared Errors (MSEs) of the models that produce the best fit of the data on the WCST indices.

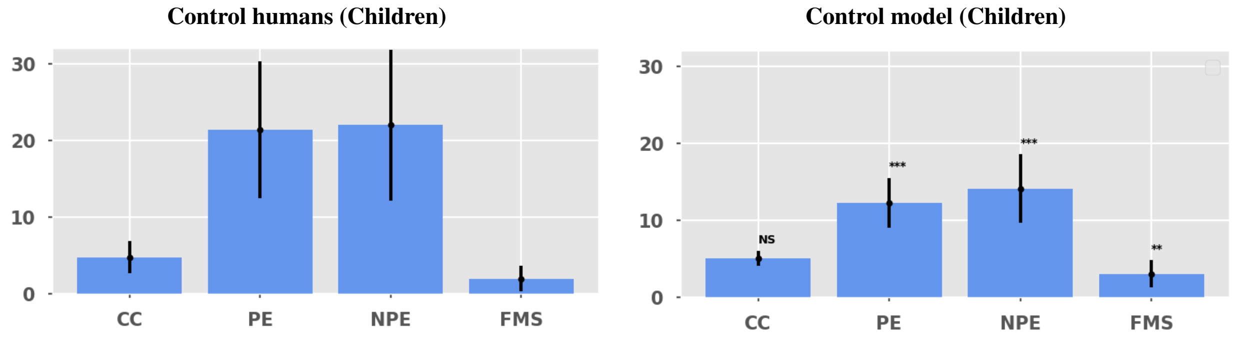

Figure S1: Children (control condition): comparison between the control model group and the control human group of children (** indicates a statistical significance of $p < 0.01$).

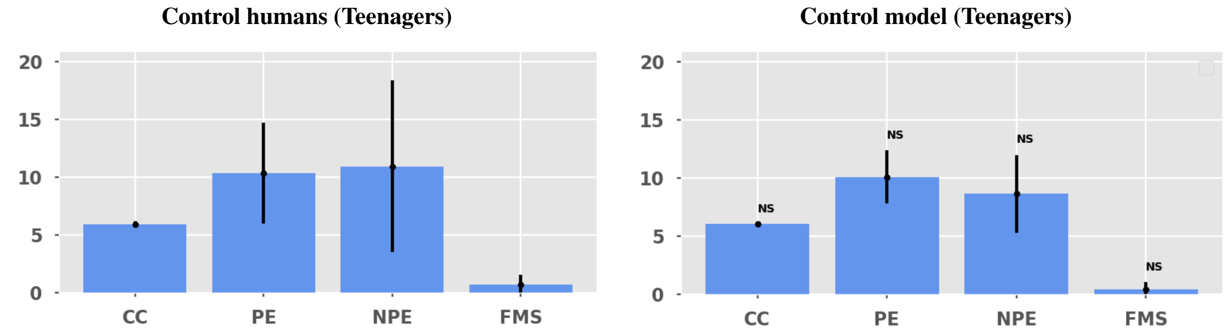

Figure S2: Teenagers (control condition): comparison between the control model group and the control human group of teenagers (** indicates a statistical significance of $p < 0.01$).

3.2 Comparison between the behaviour of different age groups (intra-condition analysis): post-hoc tables

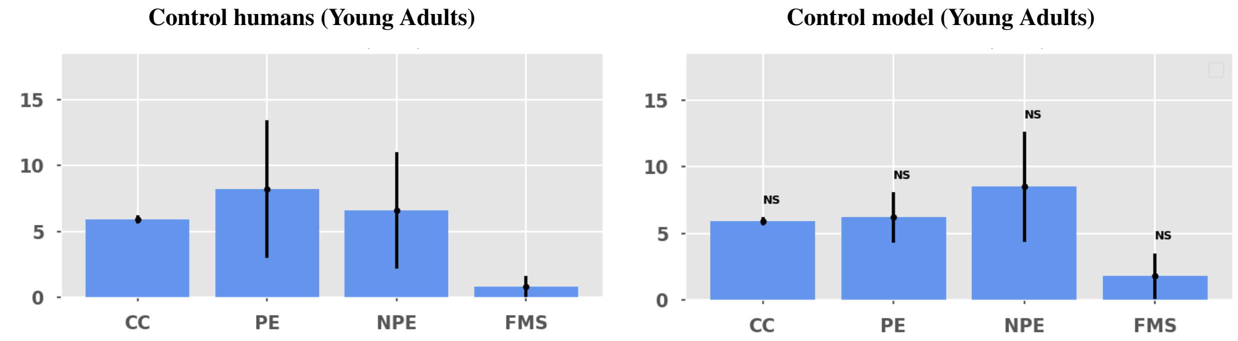

Figure S3: Young Adults (control condition): comparison between the control model group and the control human group of young adults (** indicates a statistical significance of $p < 0.01$).

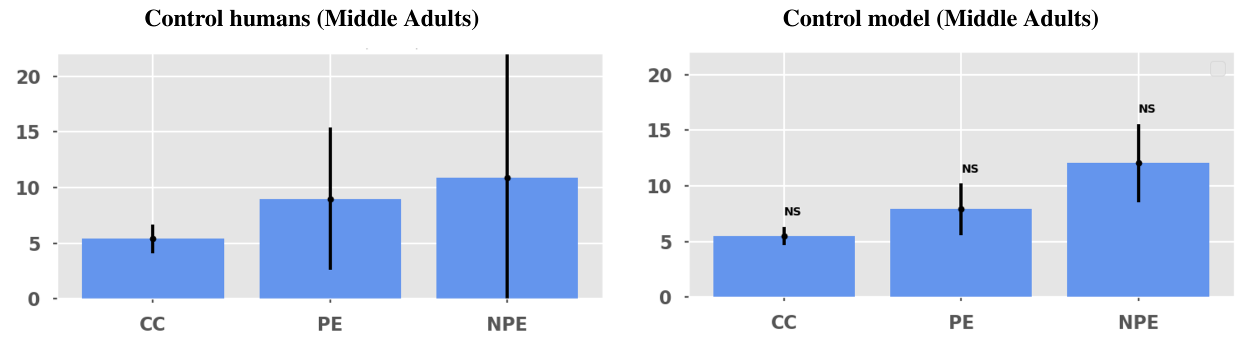

Figure S4: Middle adults (control condition): comparison between the control model group and the control human group of middle adults (** indicates a statistical significance of $p < 0.01$).

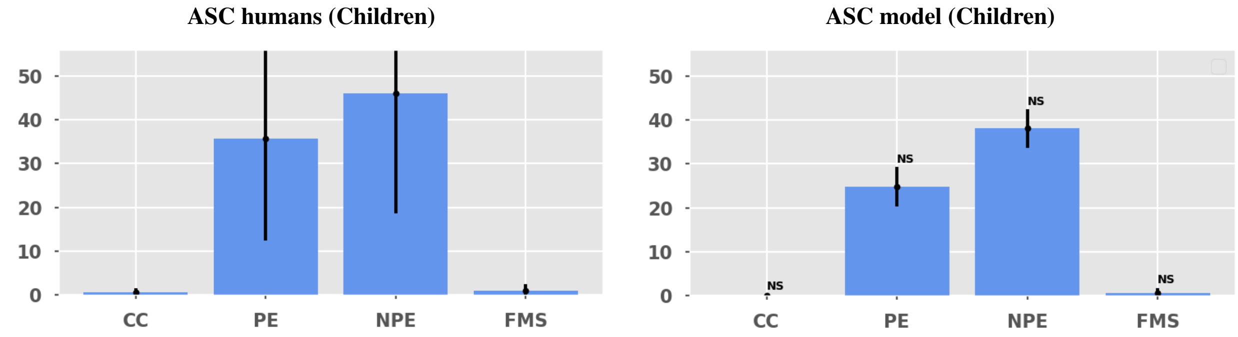

Figure S5: Children (ASC condition): comparison between the autism spectrum condition model group and the Asperger human group of children (** indicates a statistical significance of $p < 0.01$).

Post-hoc tests (CC, control condition)

	Children	Teenagers	Young adults	Middle adults
**Children**	//	$p < 0.01$	$p > 0.05$ (NS)	$p > 0.05$ (NS)
**Teenagers**	//	//	$p > 0.05$ (NS)	$p > 0.05$ (NS)
**Young adults**	//	//	//	$p > 0.05$ (NS)
**Middle adults**	//	//	//	//

Table S3: The table shows the post hoc multiple comparisons (t-test with Bonferroni correction) on CC index of control models. NS = not significant.

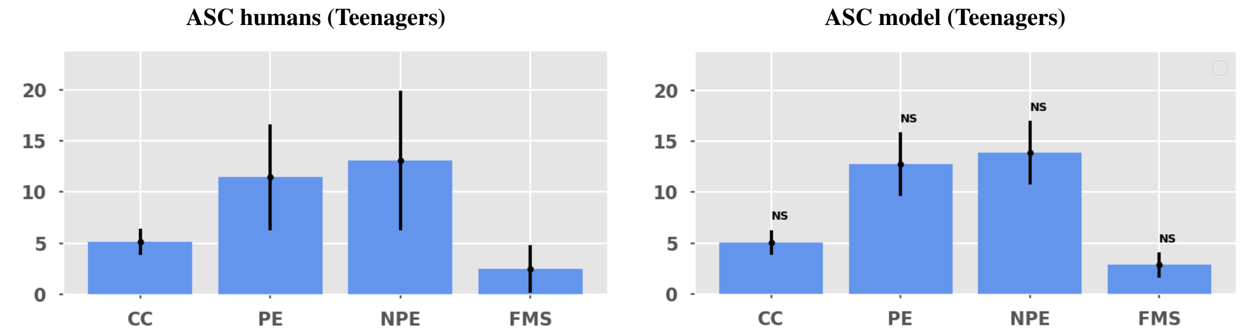

Figure S6: Teenagers (ASC condition): comparison between the autism spectrum condition model group and the a human group of teenagers (** indicates a statistical significance of $p < 0.01$).

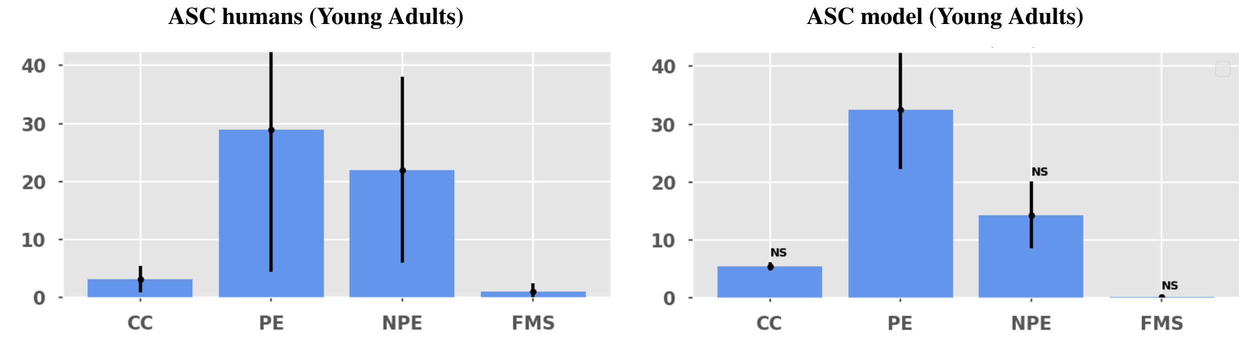

Figure S7: Young Adults (ASC condition): comparison between the autism spectrum condition model group and the a human group of young adults (** indicates a statistical significance of $p < 0.01$).

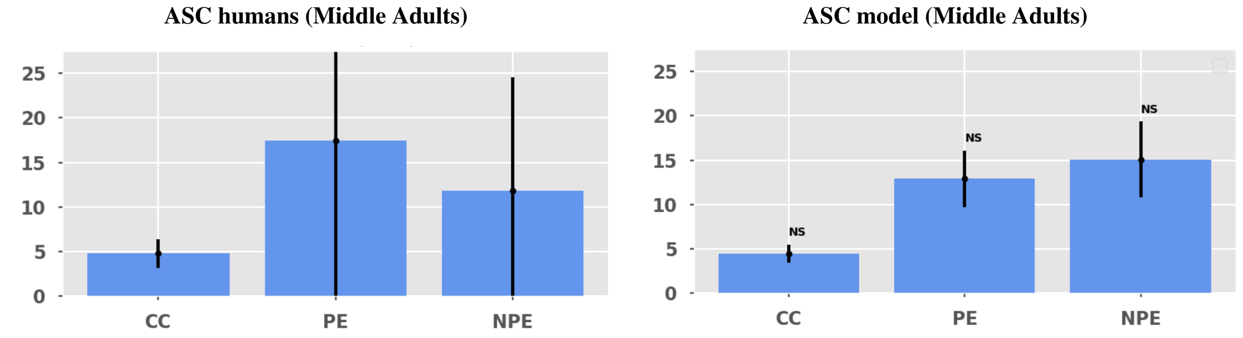

Figure S8: Middle Adults (ASC condition): comparison between the autism spectrum condition model group and the a human group of middle adults (** indicates a statistical significance of $p < 0.01$).

Post-hoc tests (CC, ASC condition)

	Children	Teenagers	Young adults	Middle adults
**Children**	//	$p < 0.001$	$p < 0.001$	$p < 0.001$
**Teenagers**	//	//	$p > 0.05$ (NS)	$p > 0.05$ (NS)
**Young adults**	//	//	//	$p > 0.05$ (NS)
**Middle adults**	//	//	//	//

Table S4: The table shows the post hoc multiple comparisons (t-test with Bonferroni correction) on CC index of ASC models. NS = not significant.

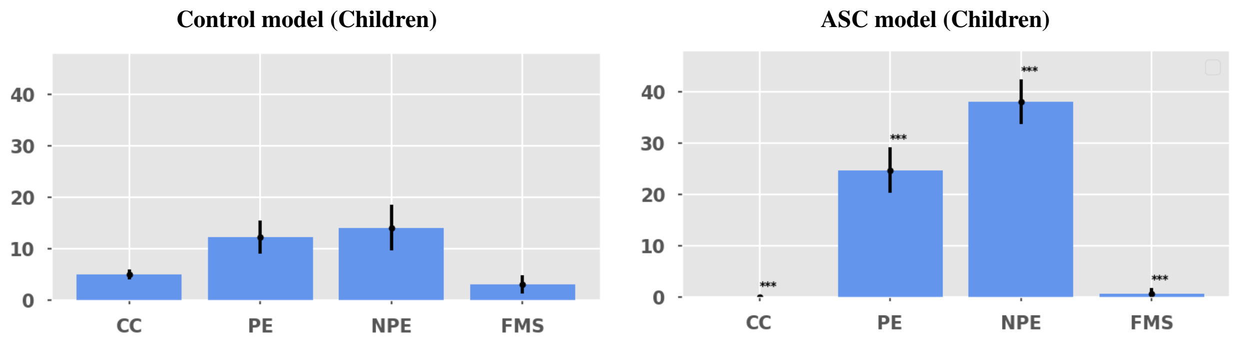

Figure S9: Children (Control-ASC conditions): comparison between the Control model and the autism spectrum condition model of children (** indicates a statistical significance of $p < 0.01$).

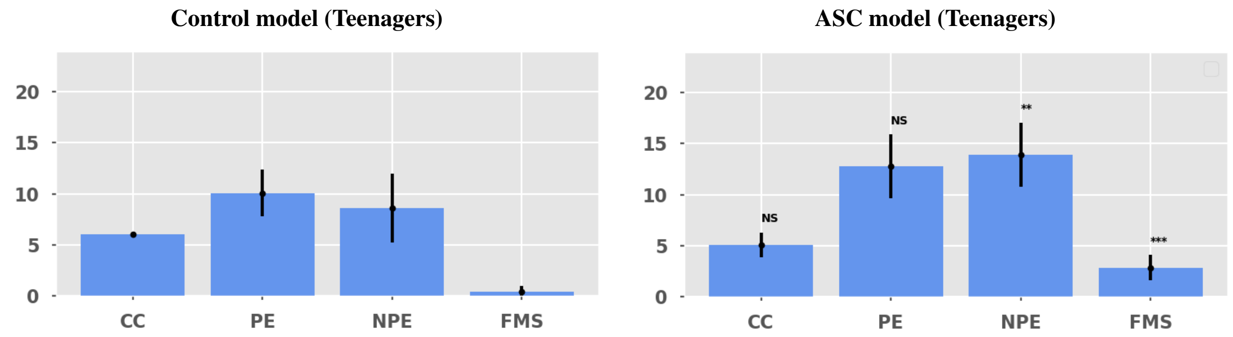

Figure S10: Teenagers (Control-ASC conditions): comparison between the Control model and the autism spectrum condition model of teenagers (** indicates a statistical significance of $p < 0.01$).

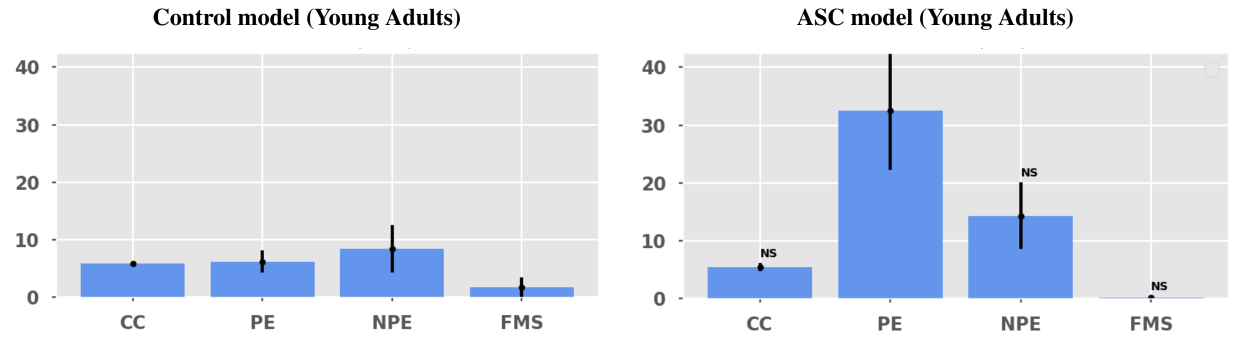

Figure S11: Young adults (Control-ASC conditions): comparison between the Control model and the autism spectrum condition model of young adults (** indicates a statistical significance of $p < 0.01$).

Post-hoc tests (PE, control condition)

	**Children**	**Teenagers**	**Young adults**	**Middle adults**
**Children**	//	$p > 0.05$ (NS)	$p < 0.001$	$p < 0.001$
**Teenagers**	//	//	$p > 0.05$ (NS)	$p > 0.05$ (NS)
**Young adults**	//	//	//	$p > 0.05$ (NS)
**Middle adults**	//	//	//	//

Table S5: The table shows the post hoc multiple comparisons (t-test with Bonferroni correction) on PE index of control models. NS = not significant.

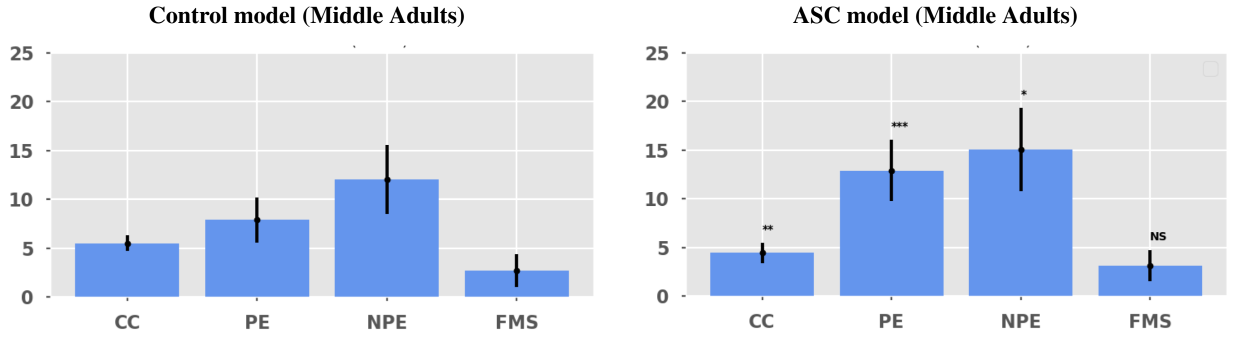

Figure S12: Middle adults (Control-ASC conditions): comparison between the Control model and the autism spectrum condition model of middle adults (** indicates a statistical significance of $p < 0.01$).

Post-hoc tests (PE, ASC condition)

	Children	Teenagers	Young adults	Middle adults
**Children**	//	$p < 0.001$	$p < 0.001$	$p < 0.001$
**Teenagers**	//	//	$p < 0.001$	$p > 0.05$ (NS)
**Young adults**	//	//	//	$p < 0.001$
**Middle adults**	//	//	//	//

Table S6: The table shows the post hoc multiple comparisons (t-test with Bonferroni correction) on PE index of ASC models. NS = not significant.

Post-hoc tests (NPE, control condition)

	Children	Teenagers	Young adults	Middle adults
**Children**	//	$p < 0.001$	$p < 0.01$	$p > 0.05$ (NS)
**Teenagers**	//	//	$p > 0.05$ (NS)	$p > 0.05$ (NS)
**Young adults**	//	//	//	$p > 0.05$ (NS)
**Middle adults**	//	//	//	//

Table S7: The table shows the post hoc multiple comparisons (t-test with Bonferroni correction) on NPE index of control models. NS = not significant.

Post-hoc tests (NPE, ASC condition)

	Children	Teenagers	Young adults	Middle adults
**Children**	//	$p < 0.001$	$p < 0.001$	$p < 0.001$
**Teenagers**	//	//	$p > 0.05$ (NS)	$p > 0.05$ (NS)
**Young adults**	//	//	//	$p > 0.05$ (NS)
**Middle adults**	//	//	//	//

Table S8: The table shows the post hoc multiple comparisons (t-test with Bonferroni correction) on NPE index of ASC models. NS = not significant.

Post-hoc tests (FMS, control condition)

	Children	Teenagers	Young adults	Middle adults
**Children**	//	$p < 0.001$	$p > 0.05$ (NS)	$p > 0.05$ (NS)
**Teenagers**	//	//	$p > 0.05$ (NS)	$p < 0.01$
**Young adults**	//	//	//	$p > 0.05$ (NS)
**Middle adults**	//	//	//	//

Table S9: The table shows the post hoc multiple comparisons (t-test with Bonferroni correction) on FMS index of control models. NS = not significant.

Post-hoc tests (FMS, ASC condition)

	Children	Teenagers	Young adults	Middle adults
**Children**	//	$p < 0.001$	$p > 0.05$ (NS)	$p < 0.001$
**Teenagers**	//	//	$p < 0.001$	$p > 0.05$ (NS)
**Young adults**	//	//	//	$p < 0.001$
**Middle adults**	//	//	//	//

Table S10: The table shows the post hoc multiple comparisons (t-test with Bonferroni correction) on FMS index of ASC models. NS = not significant.

References

[1] Schneider SG, Asarnow RF. A comparison of cognitive/neuropsychological impairments of nonretarded autistic and schizophrenic children. Journal of Abnormal Child Psychology. 1987;15(1):29–45.

[2] Rumsey JM, Hamburger SD. Neuropsychological findings in high-functioning men with infantile autism, residual state. Journal of clinical and experimental neuropsychology. 1988;10(2):201–221.

[3] Rumsey JM, Hamburger SD. Neuropsychological divergence of high-level autism and severe dyslexia. Journal of autism and developmental disorders. 1990;20(2):155–168.

[4] Szatmari P, Tuff L, Finlayson MAJ, Bartolucci G. Asperger's syndrome and autism: Neurocognitive aspects. Journal of the American Academy of Child & Adolescent Psychiatry. 1990;29(1):130–136.

[5] Ozonoff S, Pennington BF, Rogers SJ. Executive function deficits in high-functioning autistic individuals: relationship to theory of mind. Journal of child Psychology and Psychiatry. 1991;32(7):1081–1105.

[6] Ozonoff S. Reliability and validity of the Wisconsin card sorting test in studies of autism. Neuropsychology. 1995;9(4):491.

[7] Bennetto L, Pennington BF, Rogers SJ. Intact and impaired memory functions in autism. Child development. 1996;67(4):1816–1835.

[8] Ciesielski K, Harris RJ. Factors related to performance failure on executive tasks in autism. Child Neuropsychology. 1997;3(1):1–12.

[9] Nydén A, Gillberg C, Hjelmquist E, Heiman M. Executive function/attention deficits in boys with Asperger syndrome, attention disorder and reading/writing disorder. Autism. 1999;3(3):213–228.

[10] Goldstein G, Johnson CR, Minshew NJ. Attentional processes in autism. Journal of autism and developmental disorders. 2001;31(4):433–440.

[11] Liss M, Fein D, Allen D, Dunn M, Feinstein C, Morris R, et al. Executive functioning in high-functioning children with autism. The Journal of Child Psychology and Psychiatry and Allied Disciplines. 2001;42(2):261–270.

[12] Minshew NJ, Meyer J, Goldstein G. Abstract reasoning in autism: A disassociation between concept formation and concept identification. Neuropsychology. 2002;16(3):327.

[13] Geurts HM, Verté S, Oosterlaan J, Roeyers H, Sergeant JA. How specific are executive functioning deficits in attention deficit hyperactivity disorder and autism? Journal of child psychology and psychiatry. 2004;45(4):836–854.

[14] Tsuchiya E, Oki J, Yahara N, Fujieda K. Computerized version of the Wisconsin card sorting test in children with high-functioning autistic disorder or attention-deficit/hyperactivity disorder. Brain and Development. 2005;27(3):233–236.

[15] Lopez BR, Lincoln AJ, Ozonoff S, Lai Z. Examining the relationship between executive functions and restricted, repetitive symptoms of autistic disorder. Journal of autism and developmental disorders. 2005;35(4):445–460.

[16] Voelbel GT, Bates ME, Buckman JF, Pandina G, Hendren RL. Caudate nucleus volume and cognitive performance: Are they related in childhood psychopathology? Biological psychiatry. 2006;60(9):942–950.

[17] Winsler A, Abar B, Feder MA, Schunn CD, Rubio DA. Private speech and executive functioning among high-functioning children with autistic spectrum disorders. Journal of Autism and Developmental Disorders. 2007;37(9):1617–1635.

[18] Yang J, Zhou S, Yao S, Su L, McWhinnie C. The relationship between theory of mind and executive function in a sample of children from mainland China. Child psychiatry and human development. 2009;40(2):169–182.

[19] Kilincaslan A, Mukaddes NM, Küçükyazici GS, Gürvit H. Assessment of executive/attentional performance in Asperger's disorder. Turk Psikiyatri Dergisi. 2010;21(4):289.

[20] Sumiyoshi C, Kawakubo Y, Suga M, Sumiyoshi T, Kasai K. Impaired ability to organize information in individuals with autism spectrum disorders and their siblings. Neuroscience research. 2011;69(3):252–257.

[21] Kiep M, Spek AA. Executive functioning in men and women with an autism spectrum disorder. Autism Research. 2017;10(5):940–948.

[22] LeMonda BC, Holtzer R, Goldman S. Relationship between executive functions and motor stereotypies in children with autistic disorder. Research in Autism Spectrum Disorders. 2012;6(3):1099–1106.

[23] van Rijn S, Bierman M, Bruining H, Swaab H. Vulnerability for autism traits in boys and men with an extra X chromosome (47, XXY): the mediating role of cognitive flexibility. Journal of psychiatric research. 2012;46(10):1300–1306.

[24] Memari AH, Ziaee V, Shayestehfar M, Ghanouni P, Mansournia MA, Moshayedi P. Cognitive flexibility impairments in children with autism spectrum disorders: links to age, gender and child outcomes. Research in Developmental Disabilities. 2013;34(10):3218–3225.

[25] Park S, Park JE, Cho SC, Kim BN, Shin MS, Kim JW, et al. No association of the norepinephrine transporter gene (SLC6A2) and cognitive and behavioural phenotypes of patients with autism spectrum disorder. European archives of psychiatry and clinical neuroscience. 2014;264(6):507–515.

[26] Yeung MK, Han YM, Sze SL, Chan AS. Abnormal frontal theta oscillations underlie the cognitive flexibility deficits in children with high-functioning autism spectrum disorders. Neuropsychology. 2016;30(3):281.

[27] Miyajima M, Omiya H, Yamashita K, Miyata T, Yambe K, Matsui M, et al. The effects of cognitive remediation therapy using the frontal/executive program for autism spectrum disorder. The International Journal of Psychiatry in Medicine. 2016;51(3):223–235.

[28] Varanda CdA, Fernandes FDM. Cognitive flexibility training intervention among children with autism: a longitudinal study. Psicologia: Reflexão e Crítica. 2017;30.

[29] Westwood H, Mandy W, Tchanturia K. The association between symptoms of autism and neuropsychological performance in females with Anorexia Nervosa. Psychiatry Research. 2017;258:531–537.

[30] Wang Z, Jing J, Igarashi K, Fan L, Yang S, Li Y, et al. Executive function predicts the visuospatial working memory in autism spectrum disorder and attention-deficit/hyperactivity disorder. Autism Research. 2018;11(8):1148–1156.

[31] Saniee S, Pouretemad H, Zardkhaneh S. Developing set-shifting improvement tasks (SSIT) for children with high-functioning autism. Journal of Intellectual Disability Research. 2019;63(10):1207–1220.

[32] Latinus M, Cléry H, Andersson F, Bonnet-Brilhault F, Fonlupt P, Gomot M. Inflexibility in Autism Spectrum Disorder: Need for certainty and atypical emotion processing share the blame. Brain and cognition. 2019;136:103599.

[33] Lung SLM, Bertone A. Brief Report: An Exploration of Cognitive Flexibility of Autistic Adolescents with Low Intelligence Using the Wisconsin Card Sorting Task. Journal of Autism and Developmental Disorders. 2021;p. 1–7.

[34] Chen Q, Wang Z, Wan B, Chen Q, Zhai K, Jin Y. The Effect of Comorbid Attention-Deficit/Hyperactivity Disorder Symptoms on Face Memory in Children with Autism Spectrum Disorder: Insights from Transdiagnostic Profiles. Brain sciences. 2021;11(7):859.

[35] Ni HC, Lin HY, Chen YL, Hung J, Wu CT, Wu YY, et al. 5-day multi-session intermittent theta burst stimulation over bilateral posterior superior temporal sulci in adults with autism-a pilot study. Biomedical Journal. 2021;.

[36] Shu BC, Lung FW, Tien AY, Chen BC. Executive function deficits in non-retarded autistic children. Autism. 2001;5(2):165–174.

[37] Kaland N, Smith L, Mortensen EL. Brief report: cognitive flexibility and focused attention in children and adolescents with Asperger syndrome or high-functioning autism as measured on the computerized version of the Wisconsin Card Sorting Test. Journal of autism and developmental disorders. 2008;38(6):1161–1165.

[38] Rumsey JM. Conceptual problem-solving in highly verbal, nonretarded autistic men. Journal of autism and developmental disorders. 1985;15(1):23–36.

[39] Ambery FZ, Russell AJ, Perry K, Morris R, Murphy DG. Neuropsychological functioning in adults with Asperger syndrome. Autism. 2006;10(6):551–564.

[40] Hinton GE, Osindero S, Teh YW. A fast learning algorithm for deep belief nets. Neural computation. 2006;18(7):1527–1554.

[41] Hinton GE. A practical guide to training restricted Boltzmann machines. In: Neural networks: Tricks of the trade. Springer; 2012. p. 599–619.

[42] Granato G, Borghi AM, Baldassarre G. A computational model of language functions in flexible goal-directed behaviour. Scientific reports. 2020;10(1):1–13.
